# Molecular Control of the Donor/Acceptor Interface Suppresses Charge Recombination Enabling High‐Efficiency Single‐Component Organic Solar Cells

**DOI:** 10.1002/adma.202409212

**Published:** 2024-08-28

**Authors:** Yao Li, Richard A. Pacalaj, Yongmin Luo, Keren Ai, Yulong Hai, Shijie Liang, Kezhou Fan, Aleksandr A. Sergeev, Ruijie Ma, Top Archie Dela Peña, Jolanda S. Müller, Zijing Jin, P Shakya Tuladhar, Tao Jia, Jiannong Wang, Gang Li, Kam Sing Wong, Weiwei Li, James R. Durrant, Jiaying Wu

**Affiliations:** ^1^ Advanced Materials Thrust Function Hub The Hong Kong University of Science and Technology (Guangzhou) Nansha Guangzhou 511400 P. R. China; ^2^ Department of Chemistry Centre for Processable Electronics Imperial College London White City Campus London W12 0BZ UK; ^3^ Beijing Advanced Innovation Center for Soft Matter Science and Engineering & State Key Laboratory of Organic‐Inorganic Composites Beijing University of Chemical Technology Beijing 100029 P. R. China; ^4^ School of Science Department of Physics Hong Kong University of Science and Technology Clear Water Bay Kowloon Hong Kong SAR 999077 P. R. China; ^5^ Department of Electrical and Electronic Engineering Research Institute for Smart Energy (RISE) Photonic Research Institute (PRI) The Hong Kong Polytechnic University Hong Kong SAR 999077 P. R. China; ^6^ Department of Physics Imperial College London London SW7 2AZ UK; ^7^ School of Optoelectronic Engineering School of Mechanical Engineering Guangdong Polytechnic Normal University Guangzhou 510665 P. R. China; ^8^ School of Engineering Department of Chemical and Biological Engineering Hong Kong University of Science and Technology Clear Water Bay Kowloon Hong Kong SAR 999077 P. R. China

**Keywords:** double cable polymer, high‐dimensional charge transport channel, suppressed charge recombination, single‐component organic solar cells

## Abstract

Single‐component organic solar cells based on double cable polymers have achieved remarkable performance, with DCPY2 reaching a high efficiency of over 13%. In this study, DCPY2 is further optimized with an efficiency of 13.85%, maintaining a high fill factor (FF) without compromising the short circuit current. Despite its intermixed morphology, DCPY2 shows a reduced recombination rate compared to their binary counterpart (PBDB‐T:Y‐O6). This slower recombination in DCPY2 is attributed to the reduced wavefunction overlap of delocalized charges, achieved by spatially separating the donor and acceptor units with an alkyl linker, thereby restricting the recombination pathways. Adding 1,8‐diiodooctane (DIO) into DCPY2 further reduced the recombination rate by facilitating acceptor aggregation, allowing free charges to become more delocalized. The DIO‐assisted aggregation in DCPY2 (5% DIO) is evidenced by an increased pseudo‐pure domain size of Y‐O6. Fine molecular control at the donor/acceptor interface in the double‐cable polymer achieves reduced non‐geminate recombination under efficient charge generation, increased mobility, and charge carrier lifetime, thereby achieving superior performance. Nevertheless, the FF is still limited by relatively low mobility compared to the blend, suggesting the potential for further mobility improvement through enhanced higher‐dimensional packing of the double‐cable material.

## Graphical abstract

The outstanding double‐cable‐based single‐component material, DCPY2, exhibits a suppressed charge recombination process alongside efficient charge generation. This results in a high fill factor and short‐circuit current, yielding an efficiency to 13.85%. The slow recombination process in DCPY2 compared to binary systems is attributed to the reduced wavefunction overlap between donor and acceptor from inherent alkyl linker and enhanced acceptor aggregation.

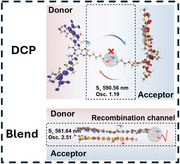

## 1 Introduction

The record power conversion efficiency (PCE) of organic solar cells (OSCs) is now approaching the 20% threshold.^[^ ^1^ ^]^ At the same time, the large‐scale commercialization of OSCs is lagging behind due to the complex deposition process of high‐performance binary systems and their thermodynamical instability under long‐term operation.^[^ ^2^ ^]^ A series of studies have shown severe phase separation of the bulk heterojunction (BHJ) under continuous light‐soaking which may be inhibited by the addition of a third component alloying with either the donor or acceptor.^[^ ^2^, ^3^, ^4^ ^]^ Another strategy to achieve morphologically stable heterojunctions is to employ single‐component films by covalently binding the donor and acceptor. This approach may also improve the reproducibility of large‐scale processing by preventing unfavorable vertical phase separation during thermal annealing.^[^ ^5^ ^]^ In this context, two types of single‐component organic solar cell (SCOSC) materials – namely conjugated block co‐polymers (BCPs) and double‐cable polymers (DCPs) – have shown great promise.^[^ ^6^, ^7^ ^]^

**Figure 1:**
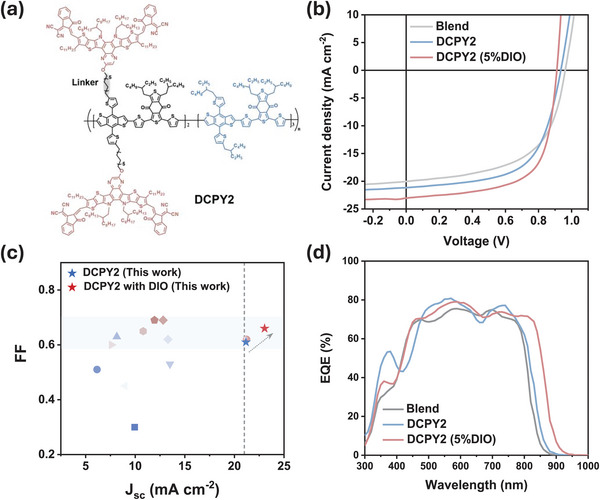
a) Chemical structure of DCPY2. b) J‐V characteristics of blend, DCPY2, and DCPY2 (5% DIO). c) Comparison of FF as a function of *J*_SC_ based on the previous double‐cable‐based single‐component materials. d) EQE spectra of blend, DCPY2, and DCPY2 (5% DIO) devices.

Double‐cable polymers consist of a conjugated donor backbone with pendent acceptors, allowing two isolated channels for electron and hole transport, respectively. Earlier DCP materials using perylene bisimide acceptors showed modest performances (< 10%), but recent DCPs incorporating Y‐type acceptors (such as DCPY2, **Figure** **1**a) have achieved high PCEs up to 13%.^[^ ^8^ ^]^ Compared to traditional BHJs, DCPs show superior stability with suppressed nanophase separation due to their covalently‐bonded donor and acceptor segments.^[^ ^6^, ^9^, ^10^, ^11^, ^12^ ^]^ However, the chemical linker between the donor and acceptor confines their aggregation behavior, leading to more intermixed domains. Consequently, the photovoltaic performance of SCOSCs may differ significantly from their binary counterparts.

In BHJs, reasonable control of nanophase separation is critical for balanced charge generation and collection. Acceptor aggregation aids the delocalization of the electron wave function, suppressing geminate recombination. Besides reducing geminate recombination, large nanophase separation also benefits charge collection by reducing non‐geminate recombination rates and facilitating charge transport in crystalline and more percolated domains. However, the over‐aggregated donor or acceptor domain would reduce donor/acceptor (D/A) interface for charge separation channel. Thus, finely controlling the donor/acceptor interface is crucial to achieve efficient charge generation while maintaining sufficient acceptor aggregation for efficient charge collection, ultimately realizing high‐performance SCOSCs with a high FF.

In binary BHJs, film morphology is typically altered by changing deposition parameters, solvent additives or post‐deposition annealing processes.^[^ ^13^, ^14^ ^]^ Also, the polymer molecular weight greatly impacts the morphology.^[^ ^15^ ^]^ Similarly, for DCPs, Feng et.al. demonstrated that under higher annealing temperature, donor backbone, and the pendent acceptor groups would self‐organize into a more ordered structure, yielding a nanophase separation of PBDBPBI‐Cl for enhanced charge transport and efficient charge generation.^[^ ^6^ ^]^ It is generally believed that 1,8‐diiodooctane (DIO) enhances acceptor aggregation, though direct observations and explanation of the role of DIO in DCPs are lacking.^[^ ^9^ ^]^ Higher molecular weight of DCP is also critical to enhance the packing order by facilitating greater interconnectivity of polymer segments.^[^ ^16^ ^]^ Previous studies on DCP morphology optimization have also focused on the molecular scale by altering the linker length or the ratio of the attached acceptor to donor backbone.^[^ ^8^, ^17^ ^]^ However, a comprehensive understanding of the structure‐morphology‐performance relationship in DCP based SCOSCs is still lacking. DCPY2 as a representative SCOSC material, shows superior performance, while its microstructure requires further analysis, and little is known about its charge carrier dynamics compared to the traditional binary systems.

Herein, we aim to understand the different photovoltaic performance of the single‐component material DCPY2 processed with and without DIO, and its binary blend counterpart (PBDB‐T: Y‐O6, Figure 1a; Figure S1, Supporting Information). DCPY2 with DIO shows a higher FF (0.66), short‐circuit current density (*J*_SC_, 23.04 mA cm^−2^), and PCE (13.85%) than the binary reference, while the open‐circuit voltage (*V*_OC_) is reduced due to the narrowing of the photovoltaic gap. Using a combination of transient optical and optoelectronic techniques, we systematically investigate the origin of the improved figures of merit. We demonstrate that the higher *J*_SC_ and FF in the SCOSCs result from efficient exciton quenching, high charge separation yield, and slow non‐geminate recombination compared to the binary blend. At the same time, their mobility remains lower than that of the blend. The addition of DIO helps acceptor aggregation and yields a mobility increase, while simultaneously improving charge separation and further reducing the non‐geminate recombination rate in DCPY2. Subsequently, the photovoltaic properties of SCOSCs and blend are connected to their morphological changes via grazing‐incidence wide & small‐angle X‐ray diffraction (GIWAXS & GISAXS) measurements complemented by density function theory (DFT) simulations. We show that the aliphatic linker acts as a spacer to suppress non‐geminate recombination kinetics by reducing the wave‐function overlap of delocalized charges between donor and acceptor moieties. The understanding of the structure‐morphology‐performance relationship in DCP‐based SCOSCs in this study will be instrumental in further enhancing their performance. By reasonably controlling the donor/acceptor interface, we can achieve efficient charge generation while maintaining reduced non‐geminate recombination. Comparing this relationship with the corresponding binary blends will aid the selection of suitable donor and acceptor components for DCPs and the rational design of the linker characteristics.

## 2 Results and Discussion

### 2.1 Understanding the Photovoltaic Performance in Single‐Component System

The binary system serves as an excellent counterpart to understanding the photophysical and morphological behavior of the state‐of‐the‐art SCOSC material DCPY2 deposited from different solvent systems. As shown in Figure 1b and Table S1 (Supporting Information), there are distinct differences in photovoltaic performance under AM 1.5G illumination between the blend, DCPY2, and DCPY2 (5% DIO). Notably, DCPY2 (5% DIO) delivered a higher PCE (13.85%) compared to DCPY2 (12.1%) and the blend (11.7%), with a high *J*_SC_ of 23.04 mA cm^−2^ and FF of 0.66 (21.16 mA cm^−2^ and 0.61 in DCPY2, 20.04 mA cm^−2^ and 0.60 in the blend), although the *V*_OC_ decreased slightly (0.911 V) compared to DCPY2 (0.934 V) and blend (0.968 V). The superior *J*_SC_ along with a high FF contributes to the improved PCE in DCPY2 (5% DIO), as seen in Figure 1c.

The absorption spectra of PBDB‐T, Y‐O6, blend, DCPY2, and DCPY2 (5% DIO) are presented in Figure S2 (Supporting Information). PBDB‐T mainly absorbs light below 650 nm, while Y‐O6 shows strong absorption from 650 to 800 nm. The absorption spectra of the blend show a broad range from the complementary absorption of donor and acceptor. Interestingly, we find that the absorption edge of Y‐O6 in the blend exactly overlaps with that of neat Y‐O6, while DCPY2 and DCPY2 (5% DIO) exhibit an absorption redshift of ≈ 20 nm. This redshift behavior from the neat acceptor has not been observed in other double cable polymers, which typically show either a blueshift or an overlapped absorption edge.^[^ ^6^, ^9^ ^]^ We propose that the origin of this redshift might be related to the different packing behavior of the acceptor (details in the discussion part). The absorption redshift of DCPY2 (5% DIO) also correlates well with the external quantum efficiency (EQE) redshift. This increased *J*_SC_ of DCPY2 (5% DIO) compared to the blend is attributed to the redshift of Y‐O6 absorption in the 800–900 nm region, which corresponds to the higher EQE in this area (Figure 1d).^[^ ^18^ ^]^ The integration of the EQE spectra yields a calculated *J*_SC_ that agrees well with the measured *J*_SC_ (Table S1, Supporting Information).

We notice that the remarkably high FF is achieved alongside a high *J*_SC_ in DCPY2, outperforming previous DCP and other intermixed binary systems. Typically, high *J*_SC_ in intermixed materials is due to the sufficient interfaces for charge separation, but it often facilitates charge recombination due to small pure domains. Therefore, it is crucial to finely regulate the donor and acceptor interface to achieve both efficient charge generation and reduced recombination. The high FF along with high *J*_SC_ in DCPY2 indicates a suppressed recombination rate even under a high population of photogenerated charges. This encouraging phenomenon drives our work toward a better understanding of the structure‐morphology‐performance relationship in DCPY2. In the following, the charge generation, non‐geminate recombination and morphology characteristics will be investigated.

### 2.2 Efficient Charge Generation in SCOSCs

**Figure 2:**
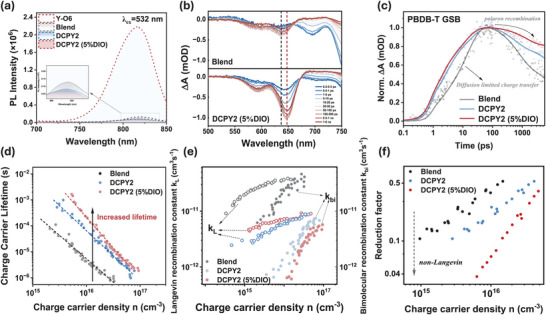
a) Steady‐state PL spectra of thin films Y‐O6, blend, DCPY2, and DCPY2 (5% DIO) under 532 nm excitation. b) Visible TAS spectra of blend and DCPY2 (5% DIO) films as a function of time delay following excitation at 800 nm under a fluence of 5 µJ cm^−2^. c) Deconvoluted PDBD‐T GSB kinetics determined from transient data shown in b), and assigned to kinetics of generation and subsequent recombination process of PDBD‐T hole polarons. d) Charge carrier lifetimes obtained from TPV measurements at open circuit as a function of charge carrier density. e) Bimolecular recombination constant determined from measured carrier lifetime (filled circles) and Langevin recombination constant determined from mobility data (open circles). f) Reduction factor determined from *k_bi_*/*k_L_* for blend, DCPY2 and DCPY2 (5% DIO) devices to investigate their non‐Langevin behavior.

**Figure** **2**a presents the steady‐state photoluminescence (PL) spectra for neat Y‐O6, blend, DCPY2, and DCPY2 (5% DIO) films, corrected for absorbance at the excitation wavelength of 532 nm. Significant PL quenching (PLQ > 97%) is observed for both DCPY2 and DCPY2 (5% DIO) films compared to neat Y‐O6 emission at 820 nm. The blend film shows a slightly lower quenching efficiency (PLQ = 94%), indicative of greater Y‐O6 phase segregation (see also time‐resolved PL and bias‐dependent PL data in Figures S3–S5, Supporting Information).

In order to correlate exciton quenching observed in our PL data with charge generation and recombination, we conducted ultrafast transient absorption spectroscopy (fs‐TAS) on thin films.^[^ ^19^, ^20^ ^]^ The TAS spectra for neat PBDB‐T and Y‐O6 films, shown in Figures S7 and S8 (Supporting Information), reveal ground state bleaching (GSB) peaks at 640 nm for PBDB‐T and 760 nm for Y‐O6 upon photoexcitation. Figure 2b shows the visible TAS spectra of blend and DCPY2 (5% DIO) films under 800 nm excitation, selectively exciting Y‐O6 to track the hole transfer kinetics. The TAS spectrum for the DCPY2 film without DIO is provided in Figure S9 (Supporting Information). In the DCPY2 and DCPY2 (5% DIO) films, the PBDB‐T GSB peak appears at 650 nm, red‐shifted by 10 nm compared to the neat PBDB‐T film. Similar redshifts are observed in the GSB and exciton peaks of Y‐O6 within the DCPY2 films (Figure S10, Supporting Information). These shifts align with the red‐shifted EQE spectra reported above. In contrast, the blend film does not exhibit this redshift, suggesting that DCPY2 films may undergo distinct morphological arrangements affecting their electronic environment. Detailed morphological analyses are discussed in Section 4.

In all cases, we are selectively exciting the acceptor, the presence of PBDB‐T GSB in DCPY2, DCPY2 (5% DIO) and blend films indicates efficient hole transfer from Y‐O6 to PBDB‐T. In both DCPY2 films, this PBDB‐T GSB emerges on sub‐picosecond timescales, much faster than in the blend film, indicating a faster hole transfer process. This difference is further illustrated in Figure S10 (Supporting Information), wherein the early‐time (0.2–0.5 ps) TAS spectra for DCPY2 and DCPY2 (5% DIO) show GSB features clearly overlapping with those observed for neat PBDB‐T, whilst the corresponding early time spectrum for the blend is dominated by GSB features overlapping with those observed for neat Y‐O6. To quantify the hole transfer kinetics, a global analysis (GA) algorithm was applied to deconvolute the PBDB‐T GSB kinetics, as plotted in Figure 2c. The rising time was fitted by a biexponential function $i\;=A_{1}\;\exp\left(-t/\tau_{1}\right)+\;A_{2}\exp\left(-t/\tau_{2}\right)$ (Table S2, Supporting Information). Here, τ_1_ is assigned, as previously, to exciton dissociation at the donor/acceptor (D/A) interface, while τ_2_ corresponds to hole transfer rate limited by the exciton diffusion time to the interface. *A*_1_ and *A*_2_ indicate their respective proportions.^[^ ^15^, ^18^, ^19^ ^]^ All three films exhibit similar fast‐rising times (τ_1_ = 1.1 ps for blend, τ_1_ = 1.2 ps for DCPY2, τ_1_ = 1.0 ps for DCPY 5% DIO), implying efficient hole transfer at the D/A interface. In contrast, the τ_2_ time is shorter in DCPY2 (10 ps) and further reduced with the addition of DIO (9 ps), compared to the blend film (15 ps). Furthermore, the *A*_2_ value is significantly higher in the blend film (72%) than in DCPY2 (45%) and 5% DIO (43%) films, demonstrating hole transfer in the blend film is more limited by exciton diffusion, which is consistent with greater phase segregation in the blend film, as indicated by the PL data above.^[^ ^21^, ^22^, ^23^ ^]^ The shorter τ_2_ in DCPY2 films represents faster exciton diffusion to the D/A interface, indicative of a more intermixed morphology, resulting in faster charge generation.^[^ ^24^, ^25^ ^]^ Consistent with this faster charge generation, the long‐lived PBDB‐T polaron signals (980 nm) are more pronounced in DCPY2 films, implying more efficient charge generation (Figure S11a–c, Supporting Information). The average hole transfer time (τ_*ave*_) calculated from the weighted contributions of τ_1_ and τ_2_ are 11, 5.4, and 4.5 ps for the blend film, DCPY2 and DCPY2 (5% DIO) films, respectively.

The charge recombination kinetics are also illustrated in the decay of the PBDB‐T GSB signal in Figure 2c. The blend film exhibits the fastest decay, whereas the DCPY2 films show slower decay, indicative of slower charge recombination. The DCPY2 recombination is further decelerated with the addition of DIO. Analogous trends in decay kinetics are also seen in the near‐infrared range for polaron photoinduced absorption (Figure S11d,e, Supporting Information).^[^ ^26^, ^27^ ^]^ We note that increased molecular intermixing, as indicated for the DCPY2 films from the PL and TAS rise kinetics discussed above, normally results in faster charge recombination. It is therefore particularly striking that the DCPY2 films exhibit not only faster charge generation but also slower recombination than the blend film. As we discuss below, we attribute this to the molecular control of the D/A interface structure in the DCPY2 films.

### 2.3 Suppressed Non‐Geminate Recombination in DCPY2

To further investigate charge recombination in DCPY2 and PBDB‐T: YO6, we turn to transient optoelectronic measurements on working devices under operating conditions. At one sun short circuit conditions, non‐geminate recombination does not limit charge collection, as indicated by the near unity power law exponents of the *J*_SC_ versus light intensity plots (Figures S12 and S13, Supporting Information). To investigate the influence of non‐geminate recombination on the FF and *V*_OC_, we measured the charge carrier densities and charge carrier lifetimes using differential capacitance and transient photovoltage measurements, respectively.

We first carried out differential capacitance measurements to investigate the charge carrier density in the active layer as a function of illumination intensity at open circuit. Figure S15a (Supporting Information) shows the resulting charge carrier density (*n*) as a function of the *V*_OC_ for the three tested devices. It is apparent that the DCPY2 with DIO devices accumulate a higher charge carrier density than other devices over the light intensity range studied herein (1 to 800% of one sun equivalent illumination). The *V*_OC_ shift along the voltage axis signifies a decrease in the effective electronic bandgap for the DCPY2 devices by up to 200 meV compared to the blend, as indicated in Figure S15a (Supporting Information). Figure S15b (Supporting Information) describes semi‐logarithmic scale of *n*‐*V*_OC_ plot and the exponential fits, which reveals a significant increase of the characteristic energy (*E*_ch_) in DCPY2 with and without DIO (50 meV and 39 meV, respectively), compared to the PBDB‐T:YO6 (33 meV).

By measuring the charge carrier lifetime under the same operating conditions, we can investigate the kinetic contribution to the *V*_OC_. Figure 2d shows the charge carrier lifetime as a function of the charge carrier density determined from transient photovoltage at open circuit. The bimolecular recombination coefficient was then determined via *k_bi_* =  1/τ_*n*_ *n* and is plotted versus the charge carrier density in Figure 2e. Interestingly, at equivalent charge carrier densities, the SCOSCs exhibit significantly longer lifetimes and lower *k_bi_* than the blend. The DCPY2 device with DIO shows an additional increase in the charge carrier lifetime. These trends in recombination kinetics are in agreement with our ultrafast TAS measurements discussed above.

We note there is a large drop in *V*_OC_ (60–100 mV) from blend to DCPY2 (5% DIO) devices, as seen in Figure 1b (see also Tables S1 and S4, Supporting Information). This voltage loss correlates with the redshift of absorption spectra and EQE spectra in DCPY2 (Figure 1d; Figure S2, Supporting Information), which are indicative of smaller optical bandgaps for DCPY2 (Figure S6, Supporting Information). A similar voltage shift has also been reported in previous systems, but without further emphasis and discussion (Table S5, Supporting Information).^[^ ^6^, ^9^, ^17^, ^28^ ^]^ Our differential capacitance data indicates reduction in electronic bandgap (Δ*V*_ele_, Table S6, Supporting Information) for DCPY2 devices exceeding that expected from the shift in the optical gap. This is partly offset by voltage gain Δ*V*_kin_ resulting from the slower recombination kinetics for DCPY2 devices. These energetic and kinetic impacts on *V*_OC_ determined from our transient data show excellent quantitative agreement (within 5 mV deviation) with the *V*_OC_ shifts observed for the devices (Table S6, Supporting Information), demonstrating the validity of the measured carrier density and lifetime data.^[^ ^29^ ^]^ Figure S16 (Supporting Information) additionally shows the excellent agreement of the *V*_OC_ reconstruction from our transient data with the experimental values across the full range of light intensities studied (see SI discussion for further explanation).

To explain the observed trends in the device FF, we carried out charge extraction measurements under short circuit conditions to calculate effective mobilities as a function of charge carrier density, as shown in Figure S17 (Supporting Information). The DCPY2 devices show a reduced mobility compared to the blend device, consistent with the expected more intermixed morphologies without large pure acceptor domains. DIO results in an increase in DCPY2 mobility, which can be explained by an increase of pseudo‐pure domains of acceptor induced by DIO, as discussed below, and reported previously in a polymer:NFA system.^[^ ^30^ ^]^ In order to quantify the impact of mobility and recombination coefficients on device FF, we calculated the Langevin recombination rate constants (*k_L_*) from the mobility data, where, and compared these to the experimentally determined *k_bi_* (Figure 2e). DCPY2 with DIO shows the biggest reduction of the recombination rate relative to the Langevin limit, as also illustrated in Figure 2f, which shows the calculated Langevin reduction factors (*k_bi_*/*k_L_*). This lowest reduction factor, of ≈0.04, correlates with DCPY2 (5% DIO) devices exhibiting the highest FF. This analysis also matches well with DCPY2 (5% DIO) devices exhibiting the highest mobility‐lifetime product (Figure S18, Supporting Information), resulting from their slower bimolecular recombination as well as a higher charge mobility than DCPY2 devices without DIO. Typically, non‐Langevin behavior with reduced reduction factors are assigned to increased phase segregation.^[^ ^31^ ^]^ In the next part we will investigate the microstructure of the active layers and correlate the photovoltaic properties with structural information.

### 2.4 DIO Assists Y‐O6 Aggregation in Double‐Cable Polymer

Above, it has been shown that double‐cable‐based single‐component materials can achieve superior performance compared to their corresponding binary systems. To understand the distinct photovoltaic performance and charge carrier dynamics between the blend and DCPY2, we investigate their morphological properties, which would have an important effect on charge transport and recombination properties.

**Figure 3:**
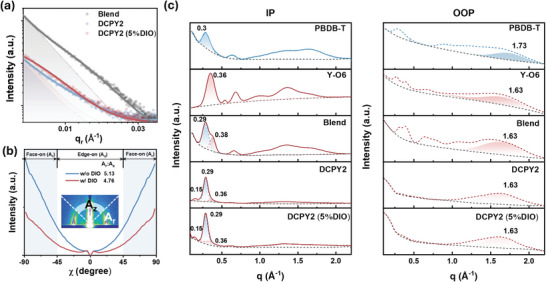
a) 1D linecuts of GISAXS profiles for blend, DCPY2, and DCPY2 (5% DIO). The solid line is the fitting curves. The dash line and shadowed area inside represent a fractal domain of the acceptor. b) Polar plot of (100) peak of DCPY2 w/o DIO, and w/ DIO, with intensity corrected by sin(χ), where χ represents polar angle. The face‐on to edge‐on orientation ratio, represented by *A_r_* (integrated area from −90 ° to −45 ° and from 45 ° to 90 °) relative to *A_z_* (integrated area from −45 ° to 45 °). c) 1D linecuts of GIWAXS plot in IP direction and OOP direction for different active layers consisting of neat PBDB‐T, neat Y‐O6, blend, DCPY2 and DCPY2 (5% DIO). Peak deconvolution for blend, DCPY2, and DCPY2 (5% DIO) is based on the neat film. Here we only focus on the analysis of lamellar stacking (100) in IP and π–π stacking (010) for the dominant face‐on packing.

According to atomic force microscopy (AFM) measurements (Figure S19, Supporting Information), DCPY2 exhibited a much smaller root‐mean‐square roughness (*R_q_*) of 0.635 nm than the blend (*R_q_* of 4.73 nm), and even smaller than the reported high‐performing binary systems.^[^ ^30^, ^32^ ^]^ This revealed the extremely delicate fiber network of DCPY2, originating from their intermixed structure. After the addition of DIO, the *R_q_* of DCPY2 increased to 0.750 nm, indicating increased phase separation from the enhanced aggregation of the Y‐O6 domain in DCPY2 (5% DIO). This is further validated by GISAXS measurements, which evaluate the domain size of donor‐ and acceptor‐rich regions (**Figure** **3**a; Figure S20, Supporting Information). It shows that DIO assists the formation of fractal‐like Y‐O6‐rich domains in DCPY2, quantified by an increased Guiniur radius (*R_q_*) from 30.8 to 36.3 nm (Table S7, Supporting Information).^[^ ^33^ ^]^ In the blend, there is a dramatic increase in scattering contrast compared to the single‐component material, with *R_q_* up to 48 nm, since the binary system allows more acceptor aggregation.^[^ ^34^ ^]^

The 2D GIWAXS plots (Figure S21, Supporting Information) demonstrates that neat Y‐O6 exhibited a highly crystalline structure, and the blend is more crystalline than the DCP films. In the blend, the formation of large domains of Y‐O6 might be due to their highly crystalline properties. The highly crystalline acceptor normally forms over‐aggregated acceptor domains that limit exciton dissociation in binary systems but are beneficial for the formation of pseudo‐pure domains to resist charge recombination in intermixed DCPs.

The 2D GIWAXS pattern of the blend shows a mixed face‐on and edge‐on orientation, while DCP shows predominantly face‐on crystallites. To quantify the orientation distribution, we calculated the ratio of face‐on crystallites (*A_r_*) and edge‐on crystallites (*A_z_*) from polar plots (Figure 3b). There is a remarkably high ratio of face‐on to edge‐on crystallites (*A_r_*/*A_z_* = 5.13) in DCPY2. The addition of DIO in DCPY2 further increases the contribution from edge‐on orientations, which agrees well with the enhanced aggregation of Y‐O6 and increased mobility (see the following discussion part). Notably, DIO induces the growth of more pseudo‐pure domains of Y‐O6 with additional edge‐on crystallites, without affecting the ordered face‐on packing, as reflected by more isotropic π–π stacking with a slightly narrower polar angle distribution (full width at half maximum, FWHM = 40.6 °) than DCPY2 without DIO (FWHM = 43.4 °) (Figure S22, Supporting Information). This indicates less face‐on misorientation and small tilt angle of π–π stacking in DCPY2 with DIO. In this case, DIO not only favors acceptor aggregation but also guarantees the ordered π–π stacking in DCPY2.

To further distinguish the different packing properties contributing to the dominant face‐on packing between DCP and binary counterparts, we plotted the 1D linecuts of films, deconvolving the lamellar stacking in in‐plane (IP) direction and π–π stacking in out‐of‐plane (OOP) direction using a Gaussian function (Figure 3c; Tables S8 and S9, Supporting Information).^[^ ^32^, ^35^, ^36^ ^]^ In the IP direction, it is clear that both donor and acceptor contribute to the lamellar stacking (100, *q* = 0.29 Å^−1^) in the blend due to the co‐existence of donor‐rich and acceptor‐rich domains (See discussion in Figure S21, Supporting Information). In contrast, the lamellar stacking from Y‐O6 is dramatically decreased in DCPY2 and DCPY2 (5% DIO) compared to the blend, due to the covalently bonded donor and acceptor unit limiting the movement of Y‐O6 in DCPY2. After DIO addition, Y‐O6 contributes more to the lamellar stacking of DCPY2 in the IP direction, indicating enhanced acceptor aggregation. In the OOP direction, all films show tight π–π stacking (010, *q* = 1.63 Å^−1^)) from Y‐O6, responsible for intermolecular electron transport.^[^ ^37^ ^]^ Overall, we constructed the microstructural framework of double‐cable polymer with the donor as polymer backbone dominantly forming lamellar stacking and the acceptor as pending side‐chain forming π–π stacking, differing from the previous reported *as*‐DCPIC where the π–π stacking comes from conjugated donor backbones.^[^ ^8^ ^]^

**Figure 4:**
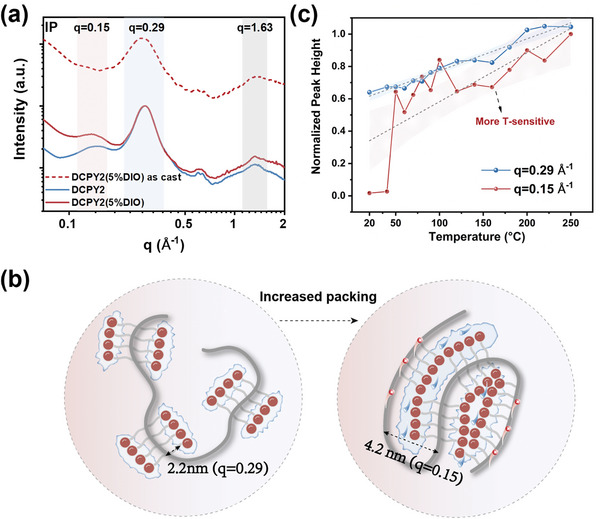
a) Comparison of lamellar stacking at *q* = 0.15 Å^−1^ in IP direction for DCPY2 w/ and w/o DIO under annealing, and for additional as‐cast DCPY2 (5% DIO) film. For DCPY2 (5% DIO) under annealing and as‐cast film, the IP linecut was normalized to the peak at *q* = 0.29 Å^−1^. b) Schematic illustration of two different kinds of packing mode of Y‐O6 in DCPY2, with GIWAXS peaks of *q* = 0.15 Å^−1^ and *q* = 0.29 Å^−1^ indicated inside. And DIO is proved to take a crucial role to increase packing of Y‐O6. c) Peak intensity of *q* = 0.29 Å^−1^ (blue) and *q* = 0.15 Å^−1^ (red) in IP direction as a function of different temperature extracted from in‐situ T‐dependent 2D GIWAXS of DCPY2 (5% DIO).

Interestingly, aside from peak at *q* = 0.29 Å^−1^, there is a novel lamellar stacking peak at *q* = 0.15 Å^−1^ in the IP direction of DCPY2 films (Figure S21e,f, Supporting Information), corresponding to a packing distance of 4.2 nm (*d* (*nm*)  =  π/5*q* (Å^−1^)), which also could be seen in other double‐cable polymers and block copolymers.^[^ ^6^, ^8^, ^17^, ^38^ ^]^ We proposed that the peak at *q* = 0.29 Å^−1^ (*d* = 2.2 nm) corresponds to the inherent donor‐acceptor distance of ≈2.2 nm, and the peak at *q* = 0.15 Å^−1^ correlates to the ordered packing between two PBDB‐T backbone, which is consistent with the distance of ≈4.2 nm between two neighboring donor chains (Figure S23, Supporting Information). To further understand the origin of this new peak, we compared the GIWAXS pattern of DCPY2, DCPY2 (5% DIO), and DCPY2(5% DIO, as cast) (**Figure** **4**a; Figure S21d–f, Supporting Information). We find that this additional peak at *q* = 0.15 Å^−1^ is present in both DCPY2 and DCPY2 (5% DIO) but disappears in the DCPY2 (5% DIO) without annealing. Although the DCPY2 (5% DIO) as‐cast film shows no peaks at *q* = 0.15 Å^−1^, there is still a strong peak at *q* = 1.63 Å^−1^ of *π–π* stacking, which means the π–π stacking primarily comes from the inherent neighboring Y‐O6 packing even without strict lamellar donor packing (Figure 4b). When two donor backbones get closer to pack in an ordered way, the peak at *q* = 0.15 Å^−1^ appears, and the intra & intermolecular π–π stacking of Y‐O6 is enhanced. This is consistent with the claim that thermal annealing can help nanophase separation of PBDBPBI‐Cl as previously discussed.^[^ ^39^ ^]^ We then investigate the role of DIO in acceptor aggregation in DCPY2. It shows that DIO enhances the scattering intensity of lamellar stacking at *q* = 0.15 Å^−1^, revealing a more tightly packed donor backbone and acceptor. This contributes to the formation of enhanced pseudo‐pure domains in double‐cable polymers to resist charge recombination and facilitate charge transport.

To further validate the assignment of the peak at *q* = 0.15 Å^−1^, we performed in‐situ temperature‐dependent GIWAXS (Figure S24, Supporting Information). Note that it is incorrect to directly compare the diffraction intensity from different samples in ex‐situ measurements. It can be seen that the peak at *q* = 0.15 Å^−1^ is more temperature‐sensitive than *q* = 0.29 Å^−1^, corresponding to previously reported temperature‐sensitive phase separation behavior (Figure 4c).^[^ ^6^ ^]^ As the temperature increases, thermally driven aggregation dominates, and donor chains tend to pack together, yielding a high diffraction density.^[^ ^6^ ^]^ Based on the above analysis, we propose that the increased pseudo‐pure domain of Y‐O6 in DCPY2 primarily comes from intra and intermolecular packing of Y‐O6.

**Figure 5:**
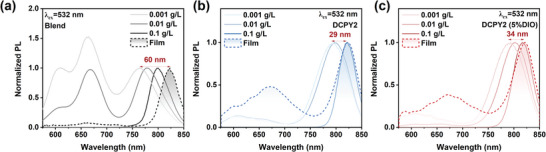
PL spectra of solution with different concentrations and neat film for a) blend, b) DCPY2, and c) DCPY2 (5% DIO).

The increased aggregation of acceptors induced by DIO could be explained by the enhanced intramolecular and intermolecular Y‐O6 packing, as discussed earlier. To prove intramolecular and intermolecular behavior in DCPY2, we compared the solution and film PL (**Figure** **5**). In the blend, there was a large redshift up to 60 nm of the PL peak from the diluted solution (0.001 mg mL^−1^) to the film due to highly aggregated free donor and acceptor units in the film. In contrast, DCPY2 and DCPY2 (5% DIO) presented a limited redshift due to the pre‐aggregated behavior in the solution, primarily from their intramolecular interaction of covalently attached Y‐O6 units, consistent with the GIWAXS results. Interestingly, the PL peak from the 0.1 mg mL^−1^ solution to the film of DCPY2 showed a negligible shift, while there was an obvious shift in DCPY2 (5% DIO), indicating more aggregated packing of Y‐O6 induced by DIO addition.

Another striking difference is the change in the relative intensity of the donor (600 to 725 nm) and acceptor (> 725 nm) PL intensity when increasing the solution concentration and finally comparing to the solid film. As the concentration of the blend solution is increased, the donor PL is quenched more efficiently relative to the acceptor PL. This is in line with the increasing interaction of the donor chains with acceptor units upon increasing solution concentrations, which leads to more efficient electron transfer while the acceptor self‐aggregation leads to a reduced acceptor PL quenching. When going from solution to the film in blend, the PL quenching of donor is most efficient due to the high proximity of donor and aggregated acceptor. In contrast to this, the intensity of the donor emission in DCPY2 increases from solution to film. This might be explained by the spatially separated donor and acceptor in DCPY2 due to the existence of chemical linker. When in solution, donor unit in DCPY2 might have higher possibility to interact with acceptor unit from another polymer backbone, enabling efficient donor PL quenching by electron transfer. In the DCPY2 films, there are small acceptor domains as evidenced from the GISAXS, enabling more efficient acceptor PL quenching in the film, which on the other hand resists the interaction between donor and acceptor by alkyl linker, thereby reducing donor PL quenching.

In conclusion, the blend exhibits the most aggregated acceptor domains that partially maintain the crystalline features of the neat Y‐O6 film. The domain size of acceptor in DCPY2, as inferred from GISAXS measurements, is increased upon the addition of DIO. The GIWAXS measurements reveal that in DCPY2, PBDB‐T backbone primarily contributes to lamellar stacking in IP direction and Y‐O6 dominates π–π stacking in OOP direction. The addition of DIO lead to a pronounced scattering peak at *q* = 0.15 Å^−1^ indicative of a lamellar packing of two donor chains, accompanied by an increase in the π–π scattering intensity of the acceptor units. The above investigations suggest that acceptor aggregation occurs between Y‐O6 units on the same polymer chain (intramolecular), as well as on lamellar stacking donor units (intermolecular).

## 3 Discussion

After analyzing the packing model of double‐cable polymer and the role of DIO additive in enhancing the acceptor‐rich domains, we now consider how these morphological changes affect the photovoltaic properties of the SCOSCs. First, the pronounced absorption redshift of DCPY2 and its origin related to the packing behavior of acceptor units will be discussed here before assessing the link between morphology and charge carrier dynamics.

Previous literature for Y‐type acceptors has highlighted that the packing type of acceptor dimers can strongly influence their electronic coupling and therefore their bandgap and absorption spectrum.^[^ ^40^, ^41^ ^]^ Compared to the packing mode of Y‐O6 in neat films or blend films with PBDB‐T, the acceptor aggregation in DCPY2 is clearly influenced by the confinement to the donor chain. The single crystal structure of Y‐O6 that has previously been published is dominated by tail‐to‐tail (TT) and core‐to‐tail (CT) interactions (illustrated in Figure S25 dimers 1, 3, and 4, Supporting Information).^[^ ^8^ ^]^ This is in contrast to the dominant dimers present in Y6 that show more molecular overlap (CT‐CT, CC‐TT) and stronger intermolecular interactions.^[^ ^41^ ^]^ The key difference to Y6 is the alkyl group on the central unit of Y‐O6 which may cause the different crystal structure through steric hindrance (Figure S1, Supporting Information).

Based on our morphological measurements for DCPY2, it seems likely that lamellar stacking donor chains (*q* = 0.15 Å^−1^) would give rise to a different intermolecular packing motif of the Y‐O6 as illustrated by dimer 2 in Figure S25 (Supporting Information). Such dimers with an increased molecular overlap may explain the redshift in the DCPY2 optical spectra due to increased intermolecular interactions as also indicated by the DFT simulations in Table S10 (Supporting Information) showing a reduced bandgap in dimer 2.^[^ ^41^ ^]^ We therefore assign the redshift in the optical spectra to the presence of these dimers with strong intermolecular interactions of the double cable polymer. These dimers may be sterically unfavorable in neat Y‐O6 or the blend. The presence of different dimers with a larger energetic distribution in DCPY2 compared to the blend would also be in line with the observation of the broader optical spectra and the increased characteristic energy (*E*_ch_) of DCPY2 (5% DIO) in the differential capacitance measurements. This may also provide an energetic gradient for electrons toward the lower bandgap regions of acceptor dimers which may affect charge generation and recombination.^[^ ^42^, ^43^ ^]^

As mentioned earlier, increased phase separation typically results in non‐Langevin behavior. However, we observe less phase separation but more non‐Langevin behavior in DCPY2 (5% DIO), compared to the blend. This indicates that the high performance of DCPY2 (5%DIO) devices cannot be understood in terms of phase separation alone.

**Figure 6:**
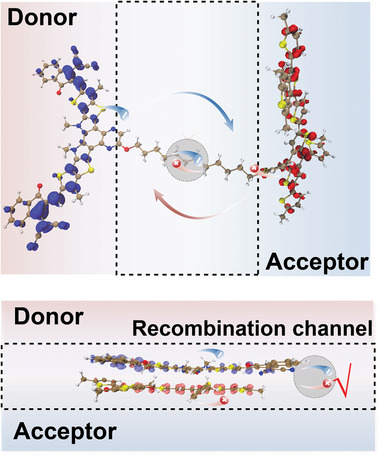
Schematic illustration of the suppressed recombination channel in DCPY2 compared to their binary counterpart.

First, we examine the reasons for more non‐Langevin behavior in DCPY2 than the blend, as reflected by a smaller reduction factor γ. The most striking structural difference between the blend and DCPY2 is the alkyl linker that covalently binds the donor and acceptor. We then assess the effect of the alkyl linker on charge recombination.^[^ ^38^, ^44^ ^]^ The physical linker in DCPY2 limits the flexible movement of acceptor units, resulting in small domains compared to the blend, while potentially blocking charge recombination channels (**Figure** **6**). Previous research has emphasized the importance of linker length in reducing non‐radiative voltage losses of DCP‐based SCOSCs.^[^ ^44^ ^]^ Here, we focus on its effect on slower recombination. After exciton separation in blend, the generated electrons in the acceptor phase may recombine with holes in the donor phase. The relative orientation and coupling between the acceptor and donor units affects the likelihood of charge recombination at the D/A interface. In double‐cable single‐component materials, the chemical linker between acceptor and donor unit may create a structural hindrance to resist recombination by reducing the electronic coupling between the donor and acceptor units. To rule out the probability of directly exciting D‐linker‐A CT states, we also simulated the electronic excited states of DCPY2 using DFT. The simulations show that either the acceptor or donor can be excited in DCPY2 (Figure S26, Supporting Information), without any charge separation from the excited states, indicating aliphatic groups in DCPY2 serves only as a physical barrier without facilitating electronic coupling between the donor and acceptor units. The reduced coupling between donor and acceptor units is illustrated in Figure 6.

Then, we consider the role of DIO in resisting Langevin behavior in DCPY2. Normally, Langevin behavior, describing bimolecular recombination, is limited by the possibility of free charge meeting another, which is highly correlated with mobility. We have demonstrated increased mobility in DCPY2 after DIO addition, attributed to increased pseudo‐pure domain. Meanwhile, a strong reduction in the bimolecular recombination rate is observed in DCPY2 with DIO. This may be due to the improved ordering observed from increased lamellar stacking of adjacent polymer chains with acceptor units intermolecularly stacking between them. This more regular morphology could aid to delocalize electrons in the more aggregated acceptor phase while further decreasing the electronic coupling with the donor chains through better alignment of the alkyl chains. The increase in mobility, along with the suppressed bimolecular recombination, results in the observed non‐Langevin behavior in DCPY2 (5% DIO) and explains the improved FF. Overall, we propose that reduced charge recombination in DCPY2 (5% DIO) is ascribed to two factors: a) the physical barrier of DCPY2 limits electronic wave‐function overlapping of donor and acceptor units, leading to a more spatially separated electron and hole that resist recombination (as illustrated in Figure 6); and b) the DIO additive further assists acceptor aggregation in DCPY2, allowing charge delocalization to overcome coulombic attraction, thus suppressing both geminate and non‐geminate recombination. Therefore, regulating the donor and acceptor interface is crucial to reduce charge recombination while maintaining efficient charge generation.

However, the reduction factor is still higher compared to the reported high FF binary systems, possibly due to the low mobility of the double‐cable polymer system.^[^ ^23^, ^31^ ^]^ To further increase mobility and photovoltaic performance of DCPs, we need to identify the bottleneck of the low mobility in DCPY2 and understand how DIO improves mobility in DCPY2.

These distinct packing ways discussed above may lead to different charge transport channels. Conventionally, face‐on orientation is favorable in high‐performance binary system due to preferred vertical charge transport.^[^ ^45^ ^]^ We show that DCPY2 has a remarkably high ratio of face‐on to edge‐on crystallites (*A_r_*/*A_z_*  = 5.13), even higher than existing high performance binary systems, indicating a negligible transport channels parallel to the substrate (Figure 3b).^[^ ^46^ ^]^ However, DCPY2 (5% DIO) presents a decreased ratio of *A_r_*/ *A_z_* = 4.76 with additional edge‐on crystallites, yet mobility is increased. We attribute this mobility increase to the formation of pseudo‐3D charge transport channels, where dominant face‐on orientation transforms into mixed face‐on and edge‐on crystallites.^[^ ^6^, ^47^ ^]^ This phenomenon is similar in previously reported BTR:Y6 and BTR‐Cl:Y6 binary systems, where introducing donors into preferential face‐on orientation of Y6 led to mixed‐orientation, therefore contributing to efficient charge transport.^[^ ^48^ ^]^

The underlying origin of this low mobility in DCPY2 is ascribed to the less phase‐separated morphology. In this respect, further investigation on thicker device of single‐component materials with high‐dimensional packing mode should be focused to increase charge mobility.

## 4 Conclusion

In this study, we comprehensively investigated the structure‐morphology‐performance relationship in high‐efficiency single‐component materials and their binary counterparts. We identified the significant effects of molecular control at the donor and acceptor interface in DCPY2 on suppressing charge recombination. The alkyl linker in DCPY2 served as chemical barrier, allowing charge delocalization at the D/A interface, thereby resisting charge recombination while still enabling effective charge generation. The addition of DIO in DCPY2 enhanced pseudo‐pure domains of Y‐O6 by increasing intra & intermolecular packing, further facilitating the delocalization of free electrons and holes. As a result, DCPY2 with DIO exhibits slower recombination and a higher FF while maintaining high *J*_SC_ even with increased charge accumulation. The efficient charge generation in DCPY2 is consistent with absorption and EQE redshift compared to the blend, resulting from energetic changes. Additionally, the molecular control through chemical linker between donor and acceptor induces the absorption redshift behavior and small optical gap of DCPY2 relative to the blend. This is because the alkyl linker confines acceptor units in the single component matrix, resulting in distinctive packing motifs differing from neat Y‐O6 structure. Despite a slight sacrifice in *V*_OC_ in DCPY2 (5% DIO), high efficiency over 13% are also received under suppressed charge recombination. The addition of DIO leads to higher mobility in DCPY2, benefiting from a transition from 2D to pseudo‐3D charge transport channels in more aggregated Y‐O6 domains. However, mobility of DCPY2 remains one order of magnitude lower than the blend, indicating the need for continued efforts in molecular control to construct high‐dimensional charge transport channels further enhance PCE.

## Conflict of Interest

The authors declare no conflict of interest.

## Author Contributions

Y.L. and R.P. contributed equally to this work. Y.L. conducted the experiments and wrote the original manuscript. J.W. proposed the research and supervised the project with J.D. Y.L. fabricated the BHJ OSCs and conducted optical and electrical characterizations. R.P. and Y.L. performed TPV/CE measurements and analyzed the data. Y.L. (Y. Luo) carried out the experiments of GISAXS/WAXS measurements. Y.H. conducted the DFT calculation. K.A. carried out Visible TAS measurements, K.F. and A.K. performed NIR TAS. J.M. carried out bias‐dependent PL. S.L. and T.J. synthesized the polymers. R.M. and P.T. optimized the devices. T.A.D.P. helped to analyze the data. J.W., G.L., K.W., and W. L. provided the experiment condition. All authors commented and revised the manuscript.

## Supporting information

Supporting Information

## Data Availability

The data that support the findings of this study are available in the supplementary material of this article.
