# Supplementary material for: Molecular Control of the Donor/Acceptor Interface Suppresses Charge Recombination Enabling High‐Efficiency Single‐Component Organic Solar Cells
Source: Adv Mater. 2024 Aug 28;37(23):2409212. doi: 10.1002/adma.202409212 (PMC12160671; doi:10.1002/adma.202409212)
Supplement: Supplementary file 1 — Supporting Information [file ADMA-37-2409212-s001.docx]

# **Molecular Control of the Donor/Acceptor Interface Suppresses Charge Recombination Enabling High-Efficiency Single-Component Organic Solar Cells**

Yao Li**,** Richard A. Pacalaj, Yongmin Luo, Keren Ai, Yulong Hai, Shijie Liang, Kezhou Fan, Aleksandr A. Sergeev, Ruijie Ma, Top Archie Dela Peña, Jolanda S. Müller, Zijing Jin, P Shakya Tuladhar, Tao Jia, Jiannong Wang, Gang Li, Kam Sing Wong, Weiwei Li, James R. Durrant, Jiaying Wu*

Y. Li, Y. Luo, Y. Hai, T.A. Dela Peña, Prof. J. Wu*

Advanced Materials Thrust, Function Hub, The Hong Kong University of Science and Technology (Guangzhou), Nansha 511400, Guangzhou, China

R. Pacalaj, K. Ai, P. Tuladhar, Prof. J. R. Durrant

Department of Chemistry, Centre for Processable Electronics, Imperial College London, White City Campus, London W12 0BZ, UK.

S. Liang, Prof. W. LI

Beijing Advanced Innovation Center for Soft Matter Science and Engineering & State Key Laboratory of Organic-Inorganic Composites, Beijing University of Chemical Technology, Beijing 100029, P. R. China

K. FAN, A. Sergeev, Z., Jin, Prof. J. Wang, Prof. K.S. Wong

School of Science, Department of Physics, Hong Kong University of Science and Technology, Clear Water Bay, Kowloon, Hong Kong SAR, P.R. China

R. Ma, Prof. G. Li

Department of Electrical and Electronic Engineering, Research Institute for Smart Energy (RISE), Photonic Research Institute (PRI), The Hong Kong Polytechnic University, Hong Kong SAR, China

J. Müller

Department of Physics, Imperial College London, London SW7 2AZ, UK.

Prof. T. Jia

School of Optoelectronic Engineering, School of Mechanical Engineering, Guangdong Polytechnic Normal University, Guangzhou, 510665, China

Prof. J. Wu*

School of Engineering, Department of Chemical and Biological Engineering, Hong Kong University of Science and Technology, Clear Water Bay, Kowloon, Hong Kong SAR, P.R. China

*Corresponding author

Contact: jiayingwu@hkust-gz.edu.cn

**Content**

Methods 4

Materials and sample preparation 4

Photovoltaic characterization 4

Morphology Characterization 4

Absorption, PL and bias-dependent PL 6

Transient absorption spectroscopy 7

Transient photovoltage and charge extraction analysis (TPV/CE) 8

DFT Calculation 9

Supplementary Reference 40

# Methods

## Materials and sample preparation

Commercial chemicals PBDB-T (from Volt-amp Optoelectronics Tech. Co., Ltd.) was used as received. The double-cable polymer and acceptor Y-O6 were prepared based on previous literature.^[1]^ Photovoltaics devices were prepared with an inverted configuration: Indium tin oxide (ITO) /ZnO/Active Layer/MoO_3_/Ag. ITO substrates were first cleaned by detergent, distilled water, acetone, isopropanol through ultra-sonication and followed by the oxygen plasma treatment. The ZnO layer is deposited by spin-coating at 4000 rpm for 40 s on the pre-treated ITO substrates. The active layer were prepared by spin-coating the solution based on DCPY2 (with 5%DIO as additive and w/o DIO) in *o*-DCB and PBDB-T:Y-O6 (1:1.2, 0.5% DIO) in CB on ZnO layer under N_2_-filled glove box, followed by annealing at 150 ℃ for 10 min and 100 ℃ for 10 min, respectively. The as-cast film is prepared without annealing process. MoO_3_ (10 nm) and Ag (100 nm) were finally deposited on thin film through thermal evaporation under vacuum. The active area of each pixel is 0.045 cm^-2^.

## Photovoltaic characterization

The *J*-*V* curves were captured by Keithley 2400 Source Meter under Newport solar simulator with AM1.5 illumination at 100 mW cm^−2^ calibrated by a certificated silicon photodiode. EQE spectra was obtained by a Solar Cell Spectral Response Measurement System QE-R3011 (Enlitech Co., Ltd.).

## Morphology Characterization

2D GISAXS/GIWAXS measurement was performed on an XEUSS 3.0 UHR SAXS/WAXS system (XENOCS, France). A Eiger2 R 1M 2-dimensional detector with 0.075 mm×0.075 mm active pixels were utilized in integration mode. The sample-to-detector distance is settled at 100/2000 mm for GIWAXS/GISAXS measurement. The precise sample-to-detector distance was determined with a silver behenate standard. The Cu incident X-ray (8 KeV) with a 0.9 mm×0.9 mm/0.5 mm×0.5 mm spot provided large enough q space. The temperature-dependent in-situ GIWAXS was performed with a dedicated temperature control stage Linkam HFSX350+RH (Linkam Scientific, Salford, United Kingdom) across a temperature range from 20 °C to 250 °C. 1D GIWAXS patterns was corrected to represent real *q_r_* and *q_z_* axis with the consideration of missing wedge. The critical incident angle was determined by the maximized scattering intensity from sample scattering with negligible contribution from underneath layer scattering. The incident angle scattering was collected at 0.2°, which renders the incident X-ray as an evanescent wave along the top surface of thin films. The samples for GIWAXS/GISAXS test were prepared by casting solution onto silicon wafer substrates (ca. 15 mm×15 mm), and the active layers were prepared using exactly the same concentration and same procedures as those for *J–V* measurements. For the polar plots, we extract the variety of scattering intensity within the changing of polar angle $\chi$. Here the polar angle “$\chi$” is the angle between the q vector and the *q_z_* component in q-space. $\chi$ is out-of-plane near 0° and in-plane near plus or minus 90°. Atomic force microscopy (AFM) of films was performed on Dimension Icon (Bruker) in a taping mode at ambient conditions.

To quantify the micro-nano structures of photovoltaic blend films, the 1D GISAXS profiles (Yonada peak, see Figure 3a) were fitted via a universal model expressed in equation 1.

$$I\left( q \right)=\frac{A_{1}}{{[1+({q\xi)}^{2}]}^{2}}+A_{2}\left( P\left( q,R \right) \right)S\left( q,R,\eta,D \right)+B (1)$$

The first term of the equation is the so-called Debye-Anderson-Brumberger (DAB) term, where *q* is the scattering wave vector, *A_1_* is an independent fitting parameter, and *ξ* is the average correlation length of the polymer-rich domain. The second term of the equation is assigned to the Fractal model, which means the occupation of fractal-like structure of the acceptor-rich domain. Notably, in our single–component system, DAB term describes the domain with more PBDB-T chain packing while fractal-liked term describes the large amount of Y-O6 packing together forming acceptor-rich domain but still maintaining a bit of PBDB-T conjugate backbone. $P(q)$ is related to the form factor of primary acceptor particles (approximated by spherical shape of radius $R$ here, and $S(q)$ is the fractal structure factor, describing the interaction between primary acceptors in this fractal-like aggregation system. The constant B is due to incoherent scattering background. $S(q)$ is given in equation 2. The η is the correlation length of the fractal-like network (or domain) formed by the aggregation of primary crystalline particles. $D$ is the fractal dimension. $R$ is the mean radius of primary crystalline particles. The domain size of this network or domain is approximately characterized by $R_{g}$, where $R_{g}$ is the Guinier radius of this fractal-like network (see equation 3).^[2]^

$$S\left( q,R,\eta,D \right)=1+\frac{\sin[\left( D-1 \right){tan}^{-1}(q\eta)}{\left( qR \right)D}\frac{D\Gamma(D-1)}{[1+\frac{1}{\left( q\eta\right)^{2}}]\frac{D-1}{2}} (2)$$

$$R_{g}=\sqrt{\frac{D(D+1)}{2}} \eta(3)$$

## Absorption, PL and bias-dependent PL

UV-Vis absorption spectra was measured by Lambda 1050 from Perkin Elmer and PL was recorded by FluoroMax 4 spectrofluorometer (Horiba Jobin Yvon). For the bias-dependent PL measurements, the device is excited with a 473 nm diode laser (Lambda Photometrics, DPBL 9010F with a Photop LDC-25005 power supply). The device emission spectrum is passed through a 595 nm long pass filter and recorded with a Shamrock 303 spectrograph combined with an iDUS InGaAs array detector (Andor SR 303i-B) cooled to -90 °C. Spectra are normalized to the detector sensitivity and the dark measurement is subtracted. The bias is applied to the device using a Keithley 2450 as voltage source.

**Time-resolved photoluminescence (TRPL)**

The sample (blend, DCPY2, DCPY2 with DIO) was excited by 800 nm using Ti:sapphire laser system with 200 fs pulses, 76 MHz repetition rate. The laser was guided and focused on the sample with a spot diameter ~1 mm by lens (focus length (f) = 250 mm). The PL signals were collected and focused on the slit by two lens (f =70 mm, f = 100 mm), and be detected by a streak camera system (Hamamatsu) with picosecond resolution. For Y-O6, the lifetime was carried out on a DeltaFlex TCSPC Lifetime spectrometer (Horiba), using a laser diode with a pump wavelength of 447 nm. All the instrument response function (IRF) was collected using quartz.

## Transient absorption spectroscopy

1. Visible TAS

The broadband pump-probe femtosecond transient absorption (TA) spectra and kinetics for thin film samples were measured using a Helios spectrometer (Spectra Physics, Newport Corp.). The setup utilized ultrafast laser pulses (800 nm, 100 fs duration) generated by a 1 kHz Ti regenerative amplifier (Solstice, Spectra Physics). A portion of these pulses was directed to an optical parametric amplifier (TOPAS Prime, Spectra-Physics) and a frequency mixer (Niruvis, Light Conversion) to create the visible pump pulses, which were modulated at 500 Hz by a mechanical chopper. The remaining 800 nm pulses were routed through a mechanical delay stage with a 6 ns time window and directed through a sapphire crystal to produce a white light probe spanning 400–900 nm in the visible region. This probe pulse was split into two beams using a neutral density filter: one beam served as the reference and was sent directly to the fiber-optic coupled spectrometers with CCD and InGaAs detectors, while the other beam, along with the pump pulse, was focused onto the sample spot (approximately 0.5 mm²). The TA measurements were conducted with the thin films placed in a quartz cuvette under a continuous nitrogen flow to ensure stability. To account for fluctuations, the spectra were normalized to the reference and averaged over multiple scans to enhance the signal-to-noise ratio. Data analysis was performed using the Surface Xplorer software.

b) Near Infrared (NIR) TAS

Transient absorption measurement was conducted via a home-built pump probe setup. A Ti:Sapphire regenerative amplifier (Coherent Legend Elite, 1000 Hz) is seeded by a Ti:Sapphire femtosecond oscillator (Coherent Mira 900; <120 fs pulse width, 76 MHz repetition rate, 800 nm central wavelength). The output of the amplifier is split into an optical parametric amplifier (Coherent Opera Solo) which provides the pump beam with tunable photon energy, while the other beam is directed onto a retroflector mounted on mechanical delay stage and subsequently focused on an yttrium aluminium garnet (YAG) plate to generate a supercontinuum probe. The pump beam is chopped at 500 Hz and incident at a small angle on the sample with a spot diameter of ~ 1.5 mm. The probe beam at a 1000 Hz repetition rate is focused onto the sample and aligned to ensure good overlap with the pump beam. Shot-by-shot transmission of the probe beam is collected by achromatic lenses analyzed in a spectrometer (Acton Spectrapro 275) with a line CCD triggered by the chopper. ΔT/T is determined by calculating the full spectrum difference in transmission of the probe pulse between pump-on and -off. The chirp of the system is corrected in software. The incident power is measured with a calibrated laser power meter.

## Transient photovoltage and charge extraction analysis (TPV/CE)

The transient optoelectronic setup is based on a circle of 12 LEDs as steady background light source to provide 0.3-10 sun equivalents controlled by a remote power supply. And the Continuum Minilite Nd:YAG laser worked as a small perturbation light source to acquire transient voltage/current signal, which is recorded by DAQ card connected to TDS3032B oscilloscope. The cell switch and light switch is controlled by the MOSFET. The one-sun equivalent light intensity is calibrated by the *J*_SC_ and *V*_OC_ obtaining from AM 1.5G solar simulator, with voltage bias applied by Keithley 2400 source meter. For the TPV measurement, the device is connected to the oscilloscope with in-put impedance of 1MΩ to ensure *V*_OC_ condition since the resistance of device is typically ~kΩ. The charge carrier density of active layer generated by the transient laser pulse is measured from charge extraction and TPV technique under open-circuit condition and then corrected by geometrical capacitance. The drift mobility is acquired from charge extraction under short circuit, where the device is connected to a 50Ω resistor to access current converted from voltage by Ohm’s law. The small perturbation lifetime $\tau_{\Delta n}$is obtained from transient voltage signal fitted using an monoexponential function and then used to estimate the total carrier lifetime $\tau_{n}$ by $\tau_{n}=\delta\tau_{\Delta n}$, where $\delta$ represents recombination order.^[3]^

## DFT Calculation

Gaussian 16 (Revision C.02) code^[4]^ was used to perform density functional theory (DFT) calculations at the non-empirically tuned B3LYP-D3(BJ)/TZVP 2-4 level of theory. The side chains of molecular were modeled as methyl groups to reduce the computational cost; while the nature of the side chains is an important factor controlling the molecule packing in the solid state, it has only marginal influence on the intrinsic electronic and optical properties of the π-conjugated backbones. In the calculation excited states, we considered a total of 50 excited states from S1 to S50 at the theoretical level of ωB97X-D /Def2tzvp and calculated the electron-hole distribution. The wavefunction software Multiwfn^[5]^ and VMD^[6]^ were used for analyzing potential energy surface (PES) and electron-hole distribution.


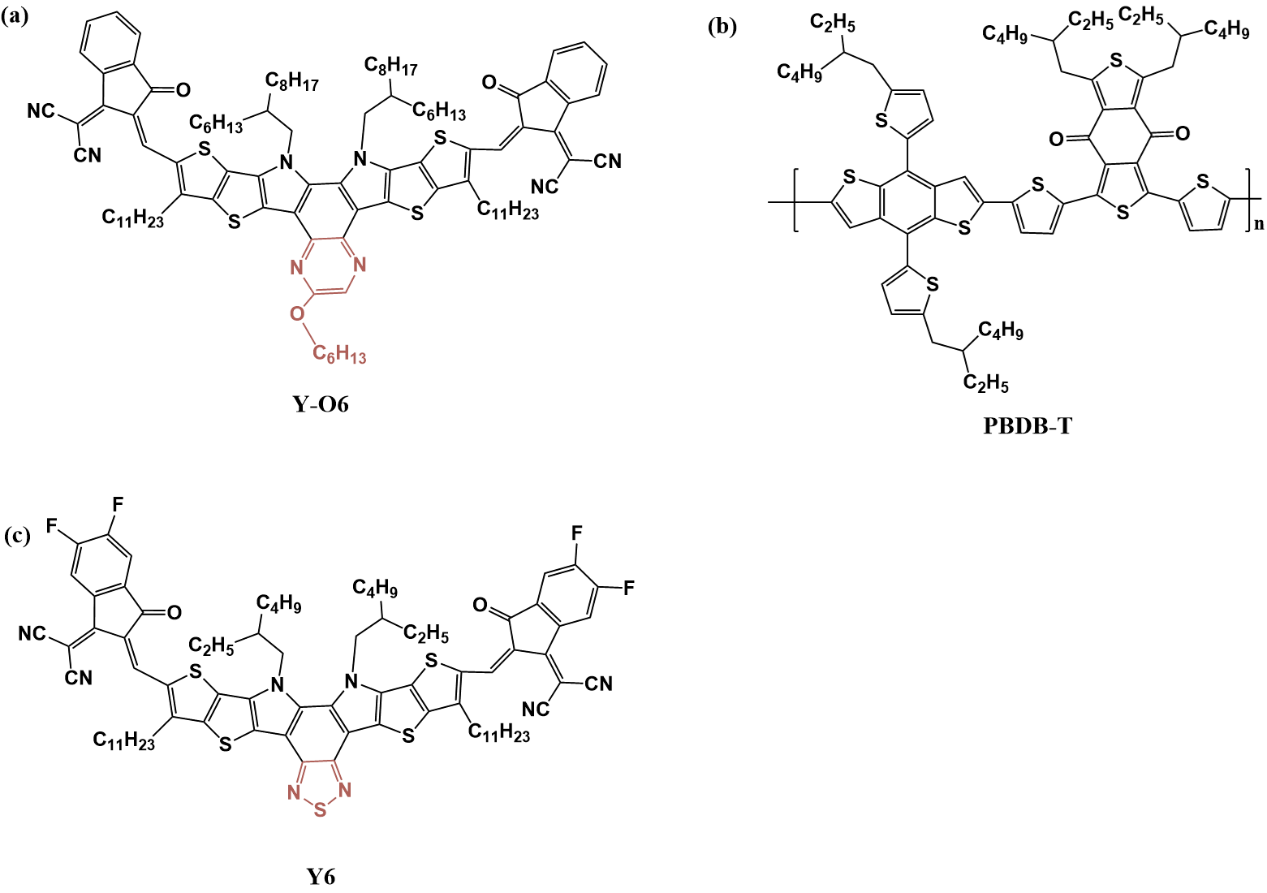


**Figure S1.** Chemical structures of a) Y-O6, b) PBDB-T and c) Y6.

# Absorption and Photoluminescence

**
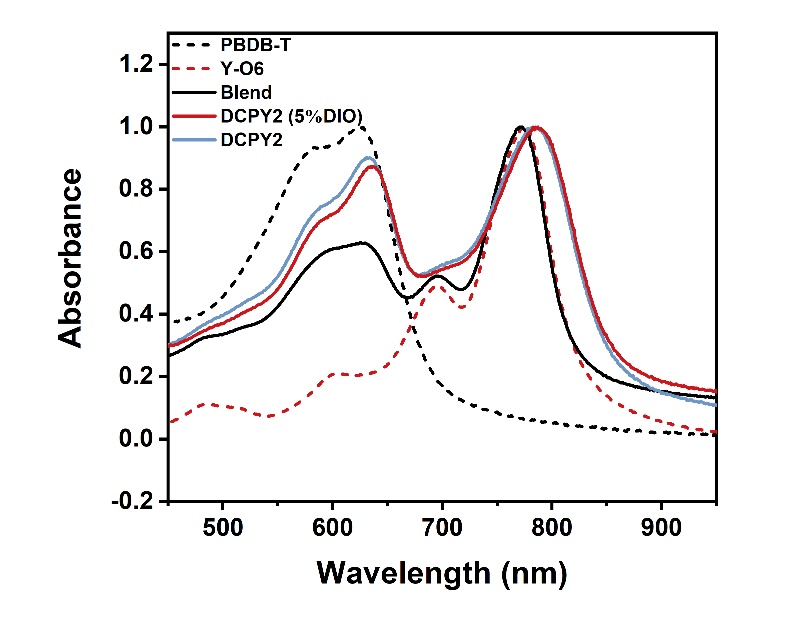
**

**Figure S2.** UV-Vis absorption spectra of PBDB-T, Y-O6, blend, DCPY2 and DCPY2 (5% DIO) thin films.


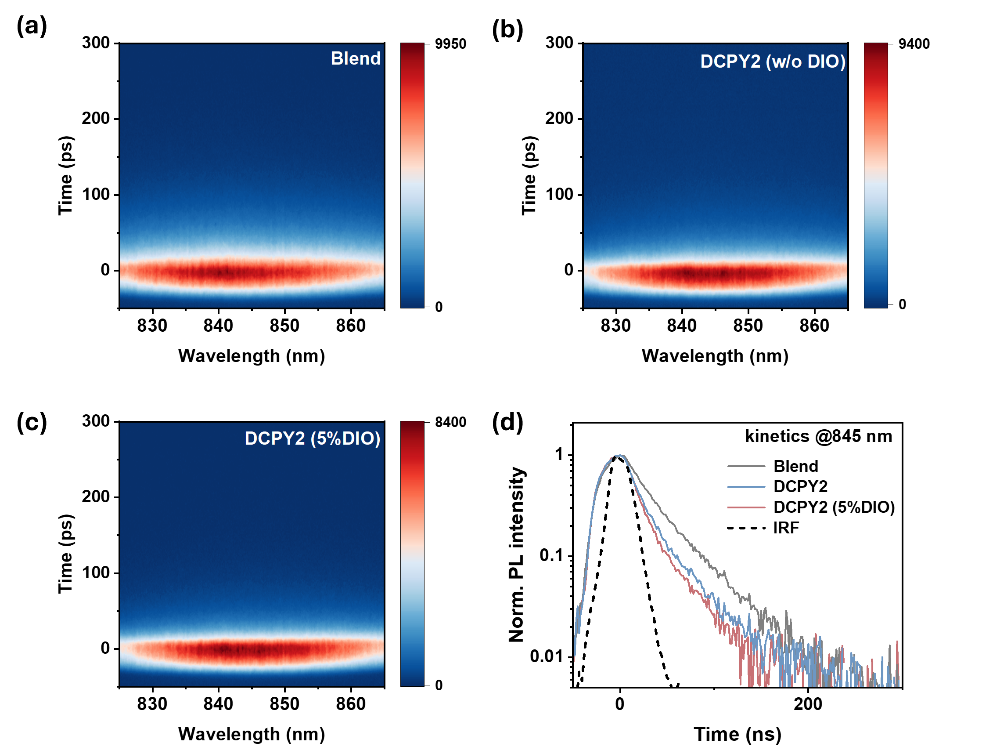


**Figure S3.** 2D Time-resolved PL (TRPL) spectra from streak camera of a) blend, b) DCPY2, c) DCPY2 (5% DIO) under excitation of 800 nm. d) 1D linecuts of TRPL kinetics probed at 845 nm.

**Figure S4.** TRPL results of Y-O6 probed at 820 nm under excitation of 477 nm.

**Figure S5.** Bias-dependent PL spectra of a) DCPY2 (5% DIO), b) DCPY2 and c) blend under reverse bias from 0 to -3 V, exciting at 473 nm. With increasing reverse bias, there is an increasing PL quenching trend observed in all three devices, indicative of the field assisted exciton separation. The PL quenching under applied bias is most pronounced in the blend, indicating that the charge separation under short circuit conditions is not complete in those devices. ^[7, 8]^ While in DCPY2 (5% DIO), there is less bias-dependent PL, which is ascribed to a sufficient internal electric field for efficient charge separation and extraction. The less bias-dependent PL quenching of the acceptor indicates an improved hole transfer process in DCPY2 (5% DIO), which is consistent with the TAS results.

# Determination of Optical gap and transport gap


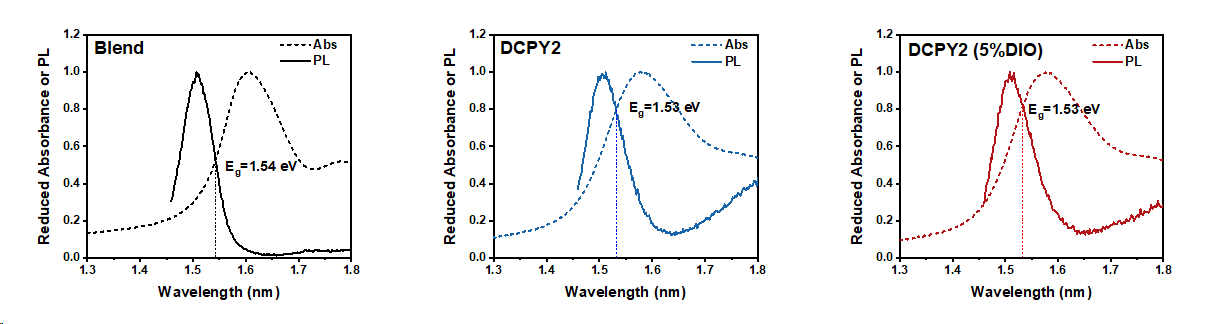


**Figure S6.** Determination of optical gap from intersection of normalized absorption spectra and PL spectra. The excitation wavelength of PL is 532 nm.

# Ultra-fast Transient Absorption Spectroscopy Measurement


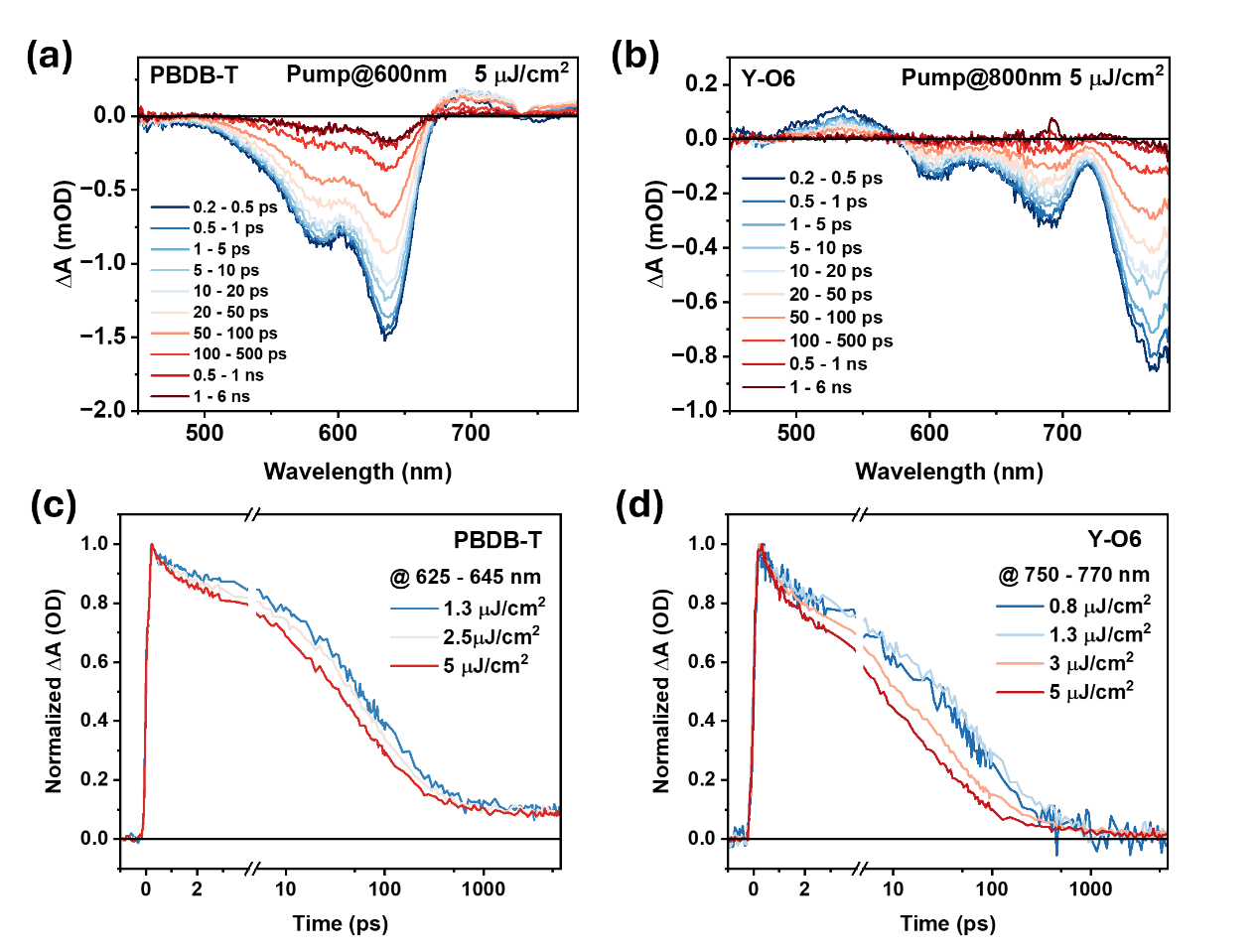


**Figure S7.** Transient absorption spectra at visible range of (a) neat PBDB-T excited at 600 nm and (b)neat Y-O6 excited at 800 nm under a fluence of 5 μJ cm^-2^. (c) The decay dynamics of neat PBDB-T probed at 625-645 nm and (d) neat Y-O6 probed at 750-770 nm. The negative peak in neat films is assigned to the GSB of its singlet exciton and the positive signal is assigned to the exciton photoinduced absorption (PIA) of neat film. After 500 ps, the majority of singlet excitons have decayed in Y-O6, while in PBDB-T film there is 10% long-live charge polarons remaining.


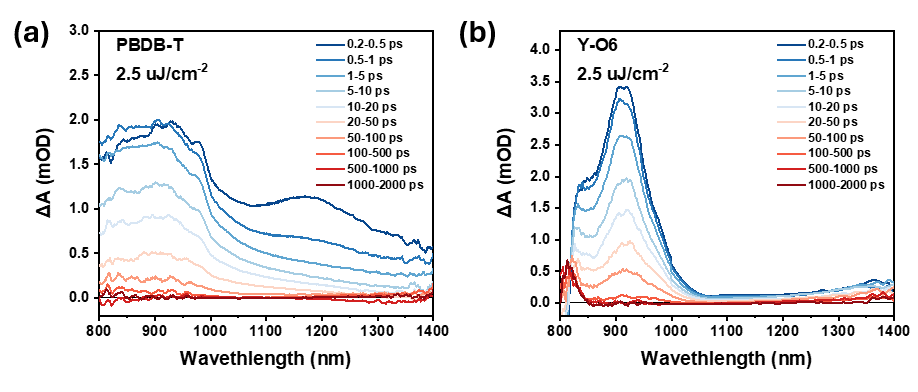


**Figure S8.** Transient absorption spectra of a) PBDB-T b) Y-O6 probed at NIR range under a fluence of 2.5 μJ cm^-2^.


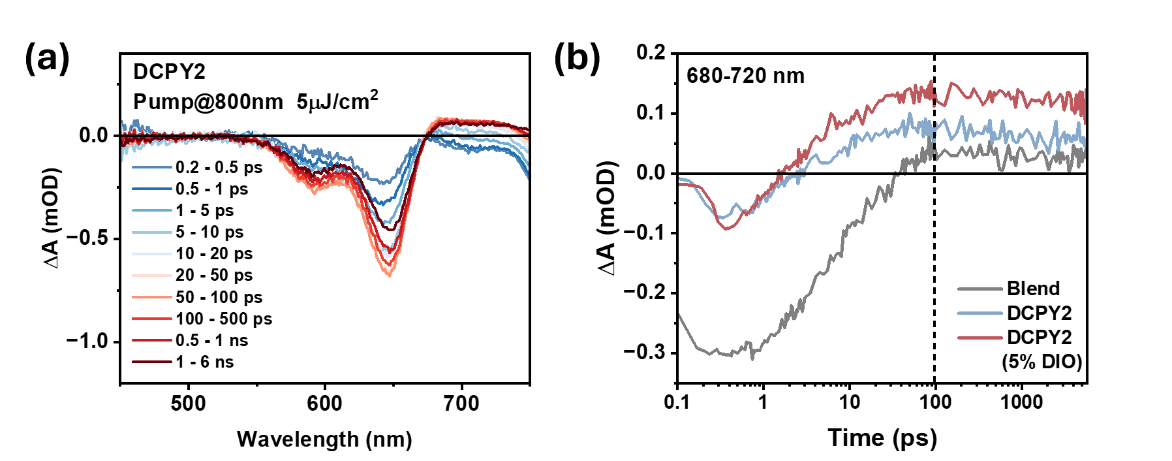


**Figure S9.** Transient absorption spectra of a) DCPY2 probed at visible range under a fluence of 5 μJ cm^-2^. The evolution of signal at 680-720 nm from negative to positive is due to the electro-absorption (EA) in PBDB-T, indicating an efficient exciton dissociation and charge generation.^[9]^ b) The decay kinetics at EA is then probed at 680-720 nm under same laser fluence 5 μJ cm^-2^. Here we only analyze the signal after 100 ps to exclude the effect from exciton PIA from donor, and it appears that DCPY2 (5% DIO) showed substantial EA signal, implying most efficient charge generation.


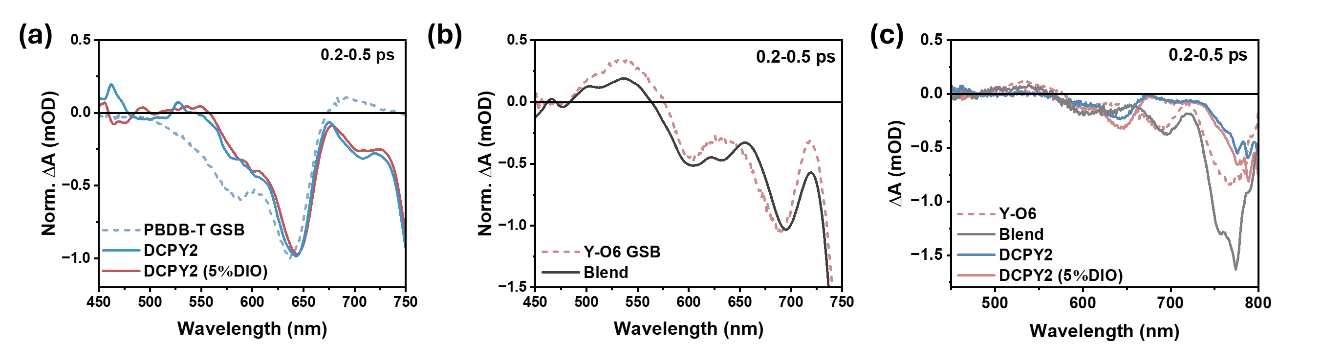


**Figure S10.** Transient absorption spectra probed at 0.2-0.5 ps of a) DCPY2 and DCPY2 (5%DIO) and b) Blend. It is clear that donor GSB appear at early time within 200 fs in DCPY2 (w and w/o DIO), while for blend, it is still dominated by acceptor Y-O6 GSB, which is ascribed to ultrafast charge transfer in DCPY2 and DCPY2 (5%DIO). c) Redshifted spectra of DCPY2 relative to blend and Y-O6 film, corresponding to the redshift absorption spectra in Figure S2.


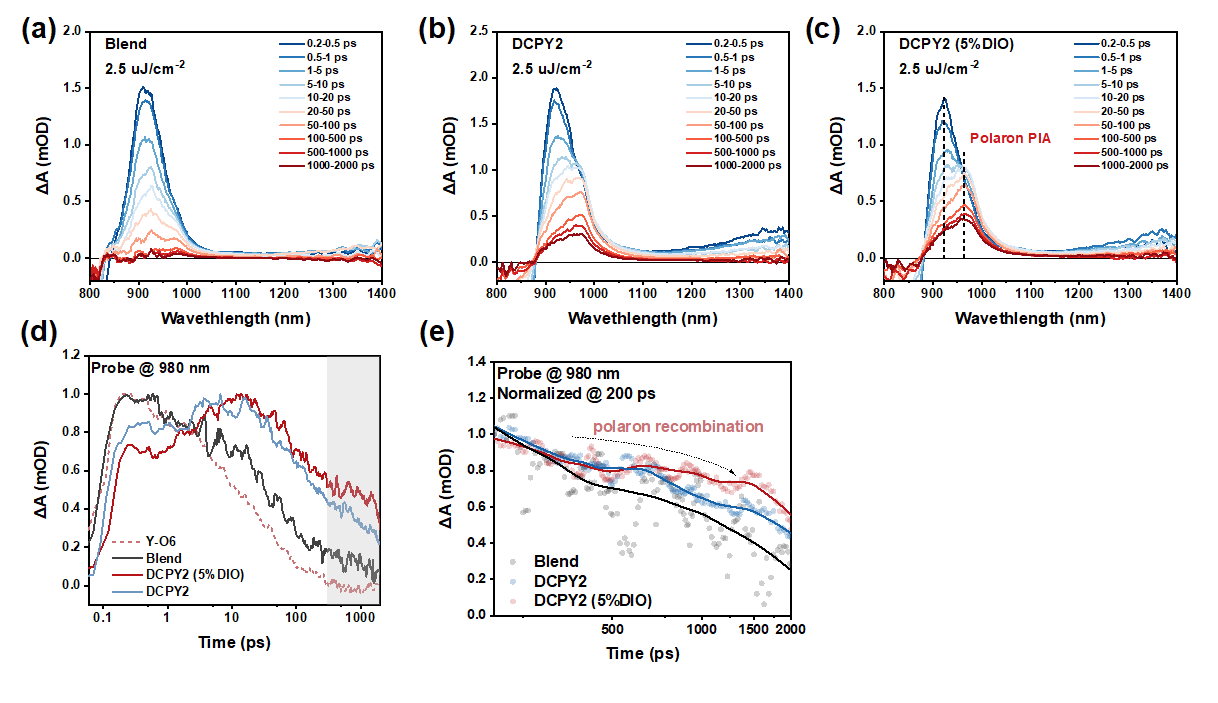
**Figure S11.** Transient absorption spectra probed at NIR range of (a) blend, (b) DCPY2 and (c) DCPY2 (5% DIO) films excited at 800 nm under a low excitation density (2.5 μJ cm^-2^). Both the DCPY2 and DCPY2 (5% DIO) films show pronounced, long-lived PIA peaks around 980 nm, which might be attributed to polaron absorption of PBDB-T. However, the polaron signal is absent in the blend films. These findings suggest both DCPY2 and DCPY2 (5% DIO) films achieve efficient charge generation, whereas the blend film shows poorer charge generation capabilities. The PIA signal of all films d) probed at 980 nm shows distinct decay behavior, where the fast decay before 200 ps is attributed to the overlap PIA signal from both Y-O6 exciton and PBDB-T polaron. To identify the polaron signal, we probe the kinetics of PIA feature e) at 980 nm after 200 ps to exclude the exciton signal from Y-O6 due to their limited exciton lifetime of neat acceptor. Clearly, the DCPY2 (5% DIO) film shows a slowest bimolecular recombination kinetics, which appeared the same trend with visible TAS results.

# TPV/CE analysis


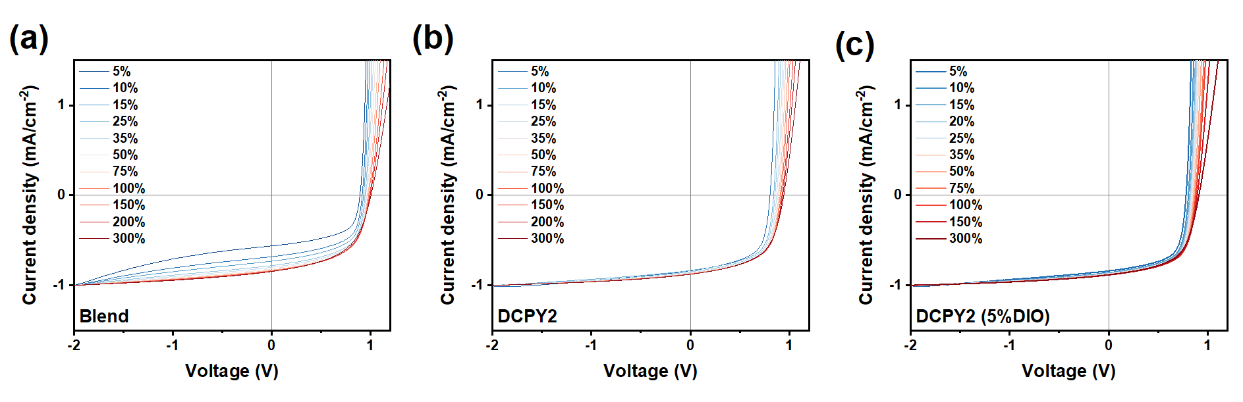


**Figure S12.** Intensity-dependent *J-V* characteristics for blend, DCPY2 and DCPY2 (5% DIO). The current density is normalized to the value under -2 V applied bias for direct comparison. The intensity-dependent *J–V* characteristics reveal that both monomolecular recombination and bimolecular recombination are suppressed in DCPY2 and the bimolecular recombination only dominated when approaching open circuit.^[10]^


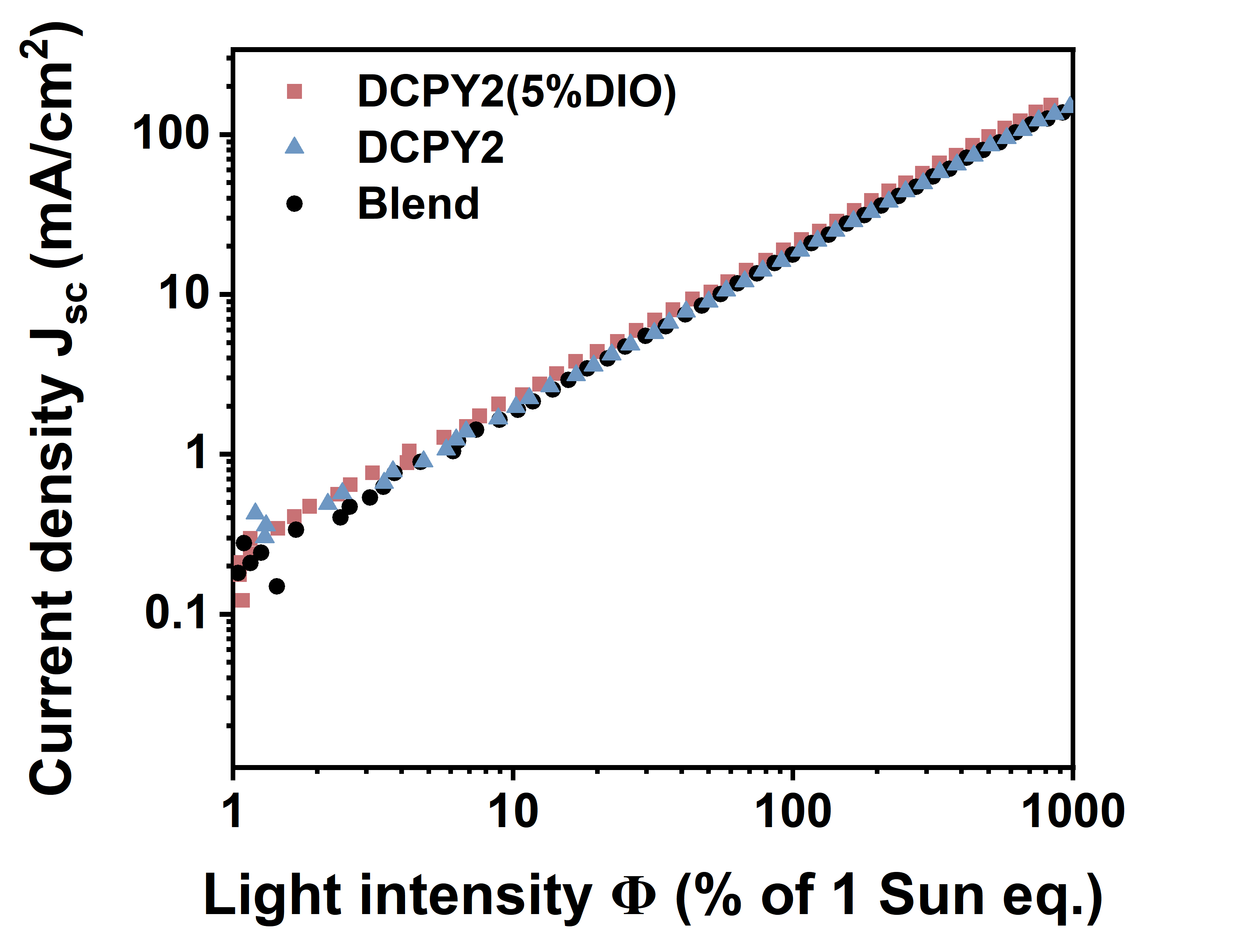


**Figure S13.** *J*_SC_ as a function of incident light intensity on a logarithmic scale.


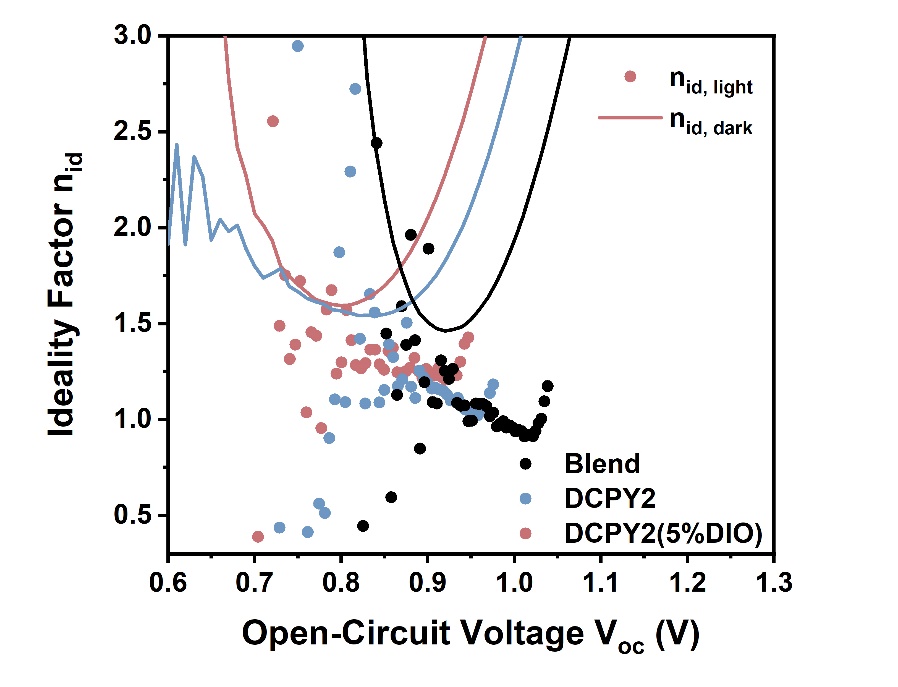


**Figure S14.** The light ideality ($n$_id, light_) and dark ideality ($n$_id, dark_) calculated from one Sun *J*-*V* curve and dark *J-V* curve, respectively.


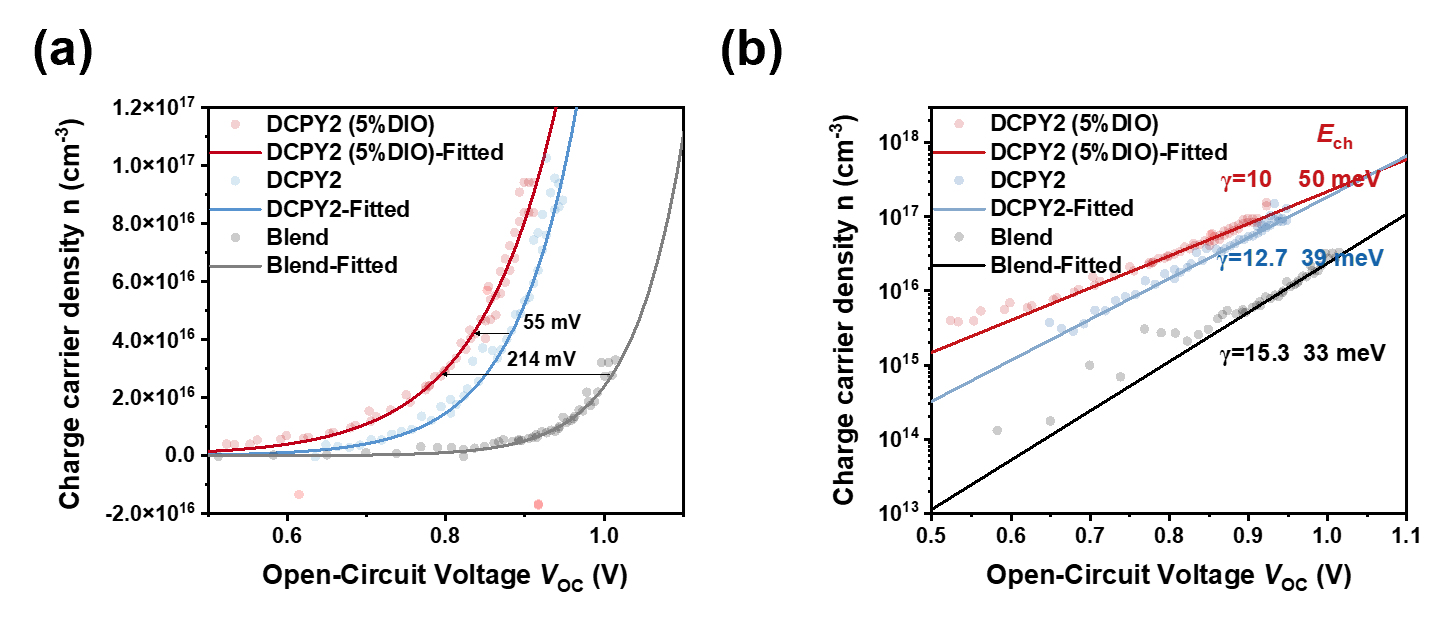


**Figure S15.** Energetic distribution of electronic states under different light intensity ($n$-*V*_OC_ plot) under a) linear scale and b) semi-log scale_._ The slope γ displayed in b) described the energetic dependence of DOS, which also indicated the Urbach energy by *E*_ch_ =1/2γ.


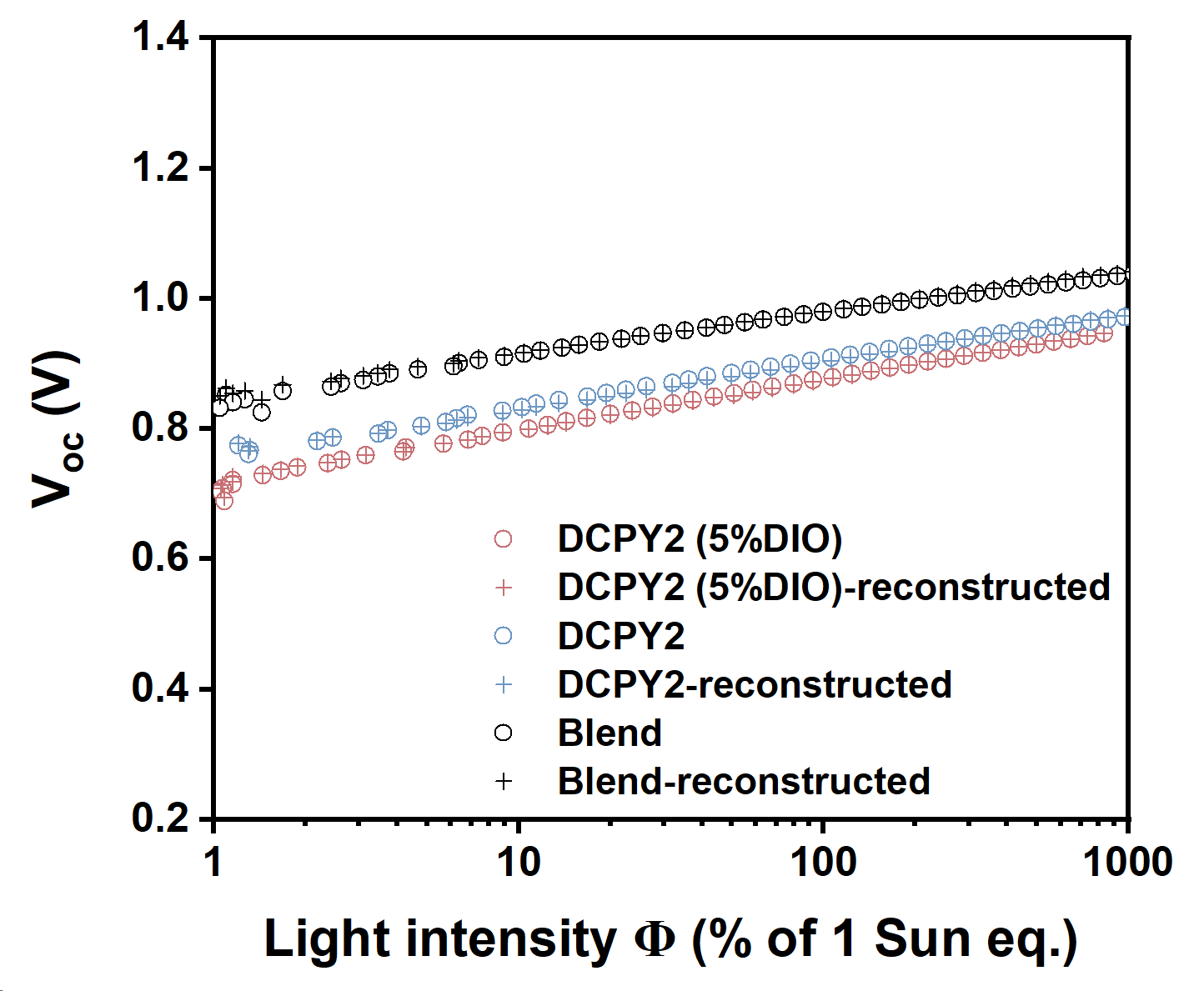


**Figure S16.** Reconstruction of *V*_OC_ under different light intensities by using experimentally determined recombination dynamics.

**Discussion on *V*_OC_ reconstruction**

At open-circuit conditions, external current *J*$\left( V_{OC} \right)$ = 0, therefore

$J_{gen}\left( V_{OC} \right)=-J_{loss}\left( V_{OC} \right)$ (4)

The device performance could be recreated if we can measure generation current and recombination current. Assuming a bias-independent charge generation process, $J_{gen}$ could be approximated by *J_SC_*. The quantification of $J_{NGloss}$ is based on the prerequisite that non-geminate recombination is the dominant loss, where $J_{loss}=edR=ed\frac{n (V_{oc})}{\tau_{n}(V_{oc})}$.

From TPV at open circuit, we can obtain the relationship between $\tau_{n}\sim V_{OC}$. In TPV measurement, a small perturbation from laser is used, therefore generating small perturbation lifetime $\tau_{\Delta n}$, which follows a single exponential decay $\tau_{\Delta n}=\tau_{\Delta n_{0}}exp(-\beta V_{OC}$). Then we should consider the relationship between small perturbation lifetime $\tau_{\Delta n_{0}}$and total charge carrier lifetime $\tau_{n}$.

In the general form of recombination kinetics, $\frac{dn}{dt}=-k_{n}n$, where pseudo-first order rate $k_{n}=k_{0}n^{\delta-1}$, $k_{0}$ is density independent constant and $\delta$ is overall recombination order.

The recombination rate of overall background with additional perturbation $\Delta n$ follows:

$\frac{d(n+\Delta n)}{dt}=-k_{0}{(n+\Delta n)}^{\delta}$ (5)

By rearranging the equation 5, we can acquire the recombination rate of small perturbation carriers:

$\frac{d\Delta n}{dt}=-\frac{dn}{dt}-k_{0}{(n+\Delta n)}^{\delta}$ (6)

Substituting $\frac{dn}{dt}=-k_{0}n^{\delta}$ in equation 6 gives:

$\frac{d\Delta n}{dt}=k_{0}n^{\delta}-k_{0}{(n+\Delta n)}^{\delta}$=$k_{0}n^{\delta}[1-\frac{\left( n+\Delta n \right)^{\delta}}{n^{\delta}}]$= $k_{0}n^{\delta}[1-(1+{\frac{\Delta n}{n})}^{\delta}]$ (7)

Assuming the small perturbation $\Delta n\ll n$, the last term could be approximated by Taylor expansion:

$(1+{\frac{\Delta n}{n})}^{\delta}\approx1+\delta\frac{\Delta n}{n}$ (8)

Combing equation 7 and equation 8, we can conclude that

$\frac{d\Delta n}{dt}\approx-\delta k_{0}n^{\delta}\Delta n$ (9)

As the extra photogenerated carriers have their own decay, following $\frac{d\Delta n}{dt}=-k_{\Delta n}\Delta n$. By substituting it into equation 9, it gives $k_{\Delta n}=\delta k_{n}$ and thus

$\tau_{n}=\delta\tau_{\Delta n}={\delta\tau}_{\Delta n_{0}}exp(-\beta V_{OC}$) (10)

As it has been proved by Shuttle, the photogenerated charges are exponentially dependent on $V_{OC}$^[11]^：

$n=n_{0}exp(\gamma V_{OC}$) (11)

Then non-geminate loss can be determined from charge carrier density from charge extraction and charge carrier lifetime from transient photovoltage at open circuit:

$J_{loss}= ed\frac{n \left( V_{oc} \right)}{\tau_{n}\left( V_{oc} \right)}=ed\frac{n_{0}\exp\left( \gamma V_{OC} \right)}{{\delta\tau}_{\Delta n_{0}}\exp\left( -\beta V_{OC} \right)}$ (12)

Therefore, $V_{OC}$ can be deducted from equation 4 and equation 12 as follows:

$V_{OC}=\frac{1}{\beta+\gamma}ln(\frac{J_{SC}\left( 1+\frac{\beta}{\gamma} \right)\tau_{\Delta n_{0}}}{edn_{0}})$ (13)


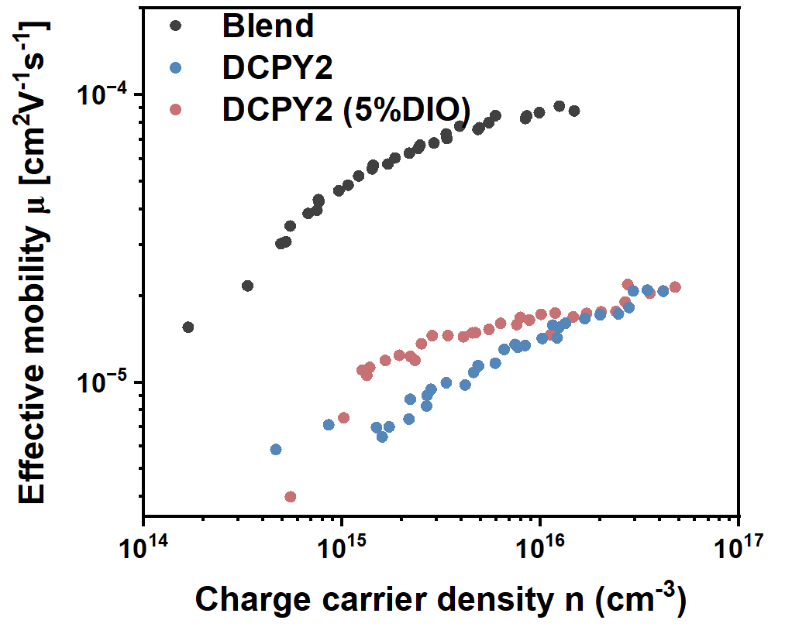


**Figure S17.** Effective mobility determined from charge extraction at short circuit versus charge carrier density.

**Calculation of mobility:**

The extraction of charge carriers in devices is related to the charge transport. Charge extraction at short circuit then can be used to calculate the drift mobility of charge carriers in devices.^[12]^ Assuming the *J_SC_* is governed by drift current,

$J_{SC}\approx J_{drift}=-e\mu_{drift}n_{SC}F\times f(\delta)$ (14)

Where $J_{drift}$is the drift current, $\mu_{drift}$ is the average drift mobility, $n_{SC}$ is the excess carrier density under different light intensity, *F* is electric field at 0 V bias, which can be assumed to be proportional to the built-in voltage $V_{bi}$ (namely, $F=\frac{V_{bi}}{d}, d$ is the active layer thickness) and $f(\delta)$ is a correction factor, which depends on recombination dynamics.

The effective mobility in devices can be calculated through

$\mu=\frac{J_{SC}*d}{e*n_{SC}}*\frac{1}{f\left( \delta\right)*V_{bi}}$ (15)


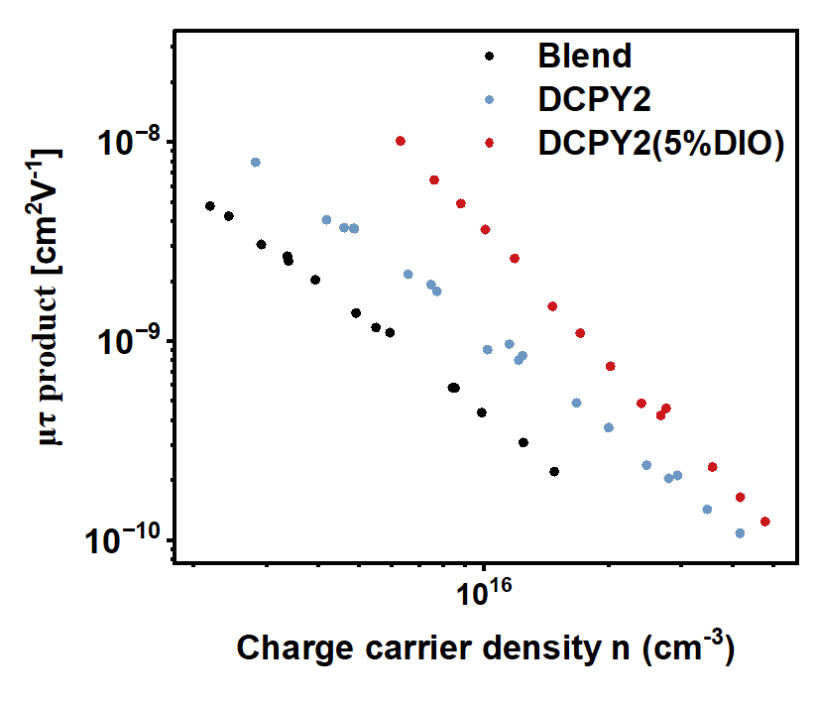


**Figure S18.** Mobility-lifetime product as a function of charge carrier density.

**Discussion on *V*_OC_ drop in DCPY2 from energetics shift:**

We find that there is large voltage drop from blend to DCPY2 (5% DIO), and similar phenomenon is also shown in other systems (Table S4-S5). To figure out the origin behind this, we comprehensively consider the individual effect of energetic contribution (charge accumulation behavior) and kinetics contribution (recombination dynamics) according to model proposed by Credgington.^[13]^

The Quantitative model to understand the origin of *V*_OC_ shift is based on the equation 16-17. And we assuming the light ideal factor $n$_id_ = 1.2 (Figure S14) based on the light-intensity *V*_OC_ measurement.^[13, 14]^

$V_{oc}=\frac{1}{q}\left( IP-EA \right)-\frac{n_{id}kT}{q}ln(\frac{J_{BI}}{J_{gen}})$ (16)

${\Delta V}_{oc}^{kin}(b-a)=-\frac{n_{id}kT}{q}ln(\frac{J_{BI}^{b}}{J_{BI}^{a}}\times\frac{J_{gen}^{b}}{J_{gen}^{a}})$ (17)

Where IP and EA denote ionization potential and electron affinity, $kT$ is thermal energy, $n$_id_ is light ideal factor, *J_BI_* and *J_gen_* denote the non-geminate loss current and generation current, respectively.

Charge extraction measurement (under open circuit) was conducted to measure the charge carrier density accumulated in the active layer under different *V*_OC_ condition enabled by changing light intensity. The measurement revealed the energetic distribution of electronic states under different charge carrier density (Figure S15), with n showing an exponential dependence on *V*_OC_, $n\propto exp(\gamma V_{oc})$,or alternatively $n\propto\Delta E_{f}/2E_{ch}$, where *V*_OC_ could be directly related to quasi-fermi level splitting (QFLS). Take *n* = 3×10^16^ cm^-3^ as example, DCPY2 showed a 159 mV drop of *V*_OC_ compared to the blend. After DIO addition, *V*_OC_ further undergo a reduction of 55 mV, resulting in an overall 227 mV of *V*_OC_ shift compared to the blend.

Then we also evaluate the impact of kinetics contribution on *V*_OC_. Charge carrier lifetime is examined by transient photovoltage at open circuit (Figure 2d). Still, take similar charge carrier density as representative, DCPY2 showed 14-fold higher lifetime compared to the blend, corresponding to the increased *V*_OC_ of 82 mV. The addition of DIO induced the 2.6-fold longer lifetime, leading to 30 mV voltage increase (Table S4). As summarized in Table S4 and Table S6, the calculated voltage shift from the in-situ measurement of energetic contribution and kinetic contribution shows remarkably good agreement with the experimental data, which further proved the dominant contribution of energetics change on voltage shift. (The approximately 5 mV deviation of *ΔV_eff_* from Δ*V*_OC_ comes from reconstruction of *V*_OC_ under different light intensities, as shown in Figure S15).

# Morphological Characterizations


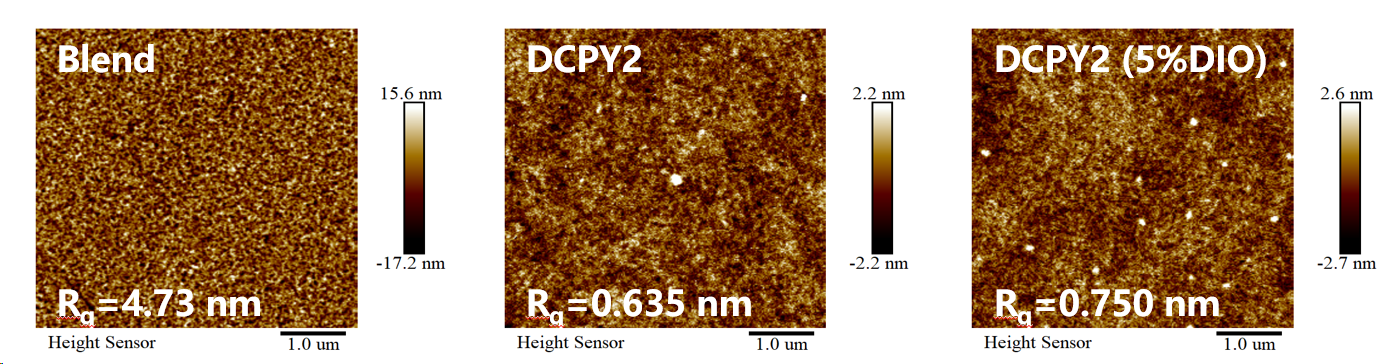


**Figure S19.** AFM images of a) blend, b) DCPY2 and c) DCPY2 (5% DIO) with root-mean-square roughness ($R_{q}$) marked inset. The surface height is recognized by the color bar.


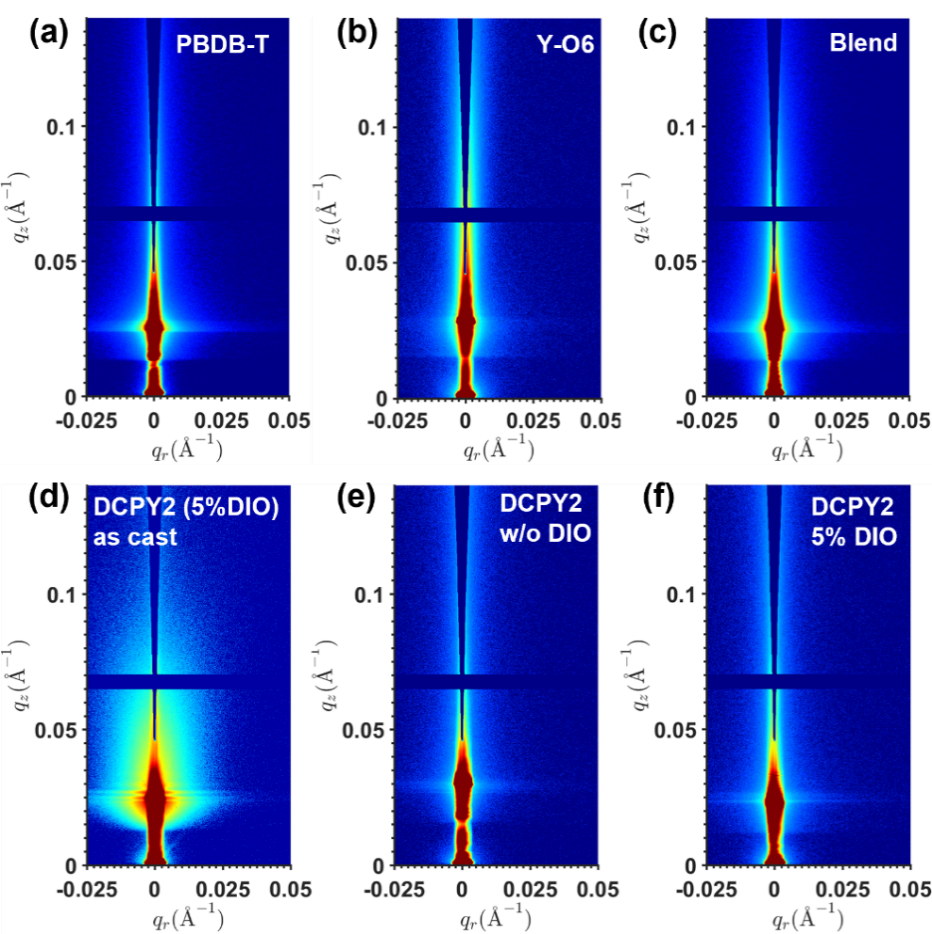


**Figure S20.** GISAXS patterns for neat a) PBDB-T, b) Y-O6, c) Blend, d) DCPY2 (5% DIO) as cast, e) DCPY2 w/o DIO and f) DCPY2 (5% DIO) under annealing. The white dash rectangle in a) indicates the integration area (Yonada peak) used to fit domain size in 1D GISAXS.


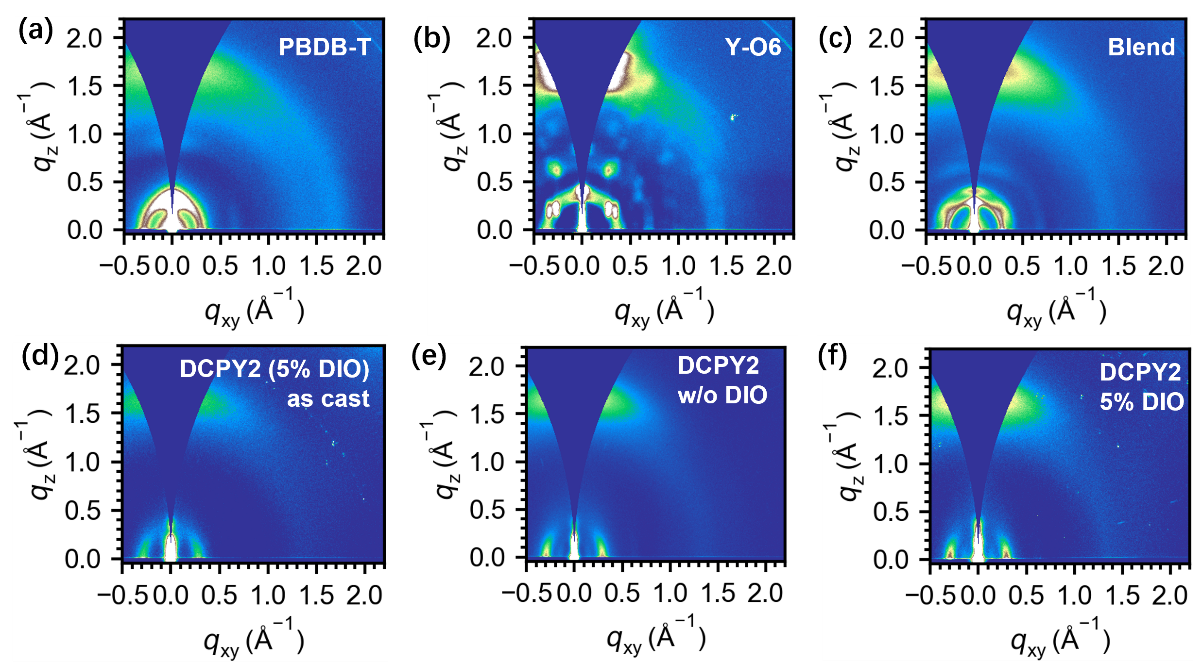


**Figure S21.** 2D GIWAXS patterns of a) PBDB-T and b) Y-O6, c) blend, d) as-cast prepared DCPY2 (5% DIO), e) DCPY2 w/o DIO and f) DCPY2 with 5% DIO annealing under 150 ℃.

**Discussion on peak assignments of GIWAXS**

The corresponding 1D linecuts are provided in Figure 3c. Here, the lamellar stacking (100) peak of pure PBDB-T and YO6 are located at *q* = 0.3 Å^-1^ (*d* = 2.1 nm) and *q* = 0.36 Å^-1^ (*d* = 1.74 nm), respectively. And the 𝜋-𝜋 stacking (010) peak of PBDB-T and YO6 are at *q* = 1.73 Å^-1^ (*d* = 0.36 nm) and *q* = 1.63 Å^-1^ (*d* = 0.39 nm). Turing to DCPY2 and blend, it is obvious that both lamellar stacking (100) of donor and acceptor contribute to the scattering in DCPY2 and blend. The (100) peak intensity of Y-O6 in blend is much larger than that of DCPY2 which might indicate the well-organized Y-O6 packing in blend but dispersed Y-O6 packing in DCPY2 as we discussed in main text. For 𝜋-𝜋 stacking (010) peak, both blend and DCPY2 shows peak at q = 1.63 Å^-1^ (*d* = 3.85 nm) from Y-O6. This result suggest that the alkyl-chain linker only break the horizontal lamellar stacking packing of Y-O6 in DCPY2 but keep vertical 𝜋-𝜋 stacking packing of Y-O6, which is supposed to be beneficial to charge transport. Notably, for the assignment of *q* = 0.29 Å^-1^ in DCPY2 and DCPY2 (5% DIO), it might originate from 1) D-A lamellar stacking structure (see Figure S23); 2) the sum of second order diffraction peak of q= 0.15 Å^-1^ and (001) peak. Normally the second order diffraction peak is much weaker than the first order and the spacing along the ring direction is too disordered to give a well-defined (001) peak. Here the intensity of *q* = 0.29 Å^-1^ is much larger than peak of *q* = 0.15 Å^-1^. We proposed the peak at *q* = 0.29 Å^-1^ may not come from reason 2 but reason 1, which is D-A lamellar stacking (see Figure 4b).


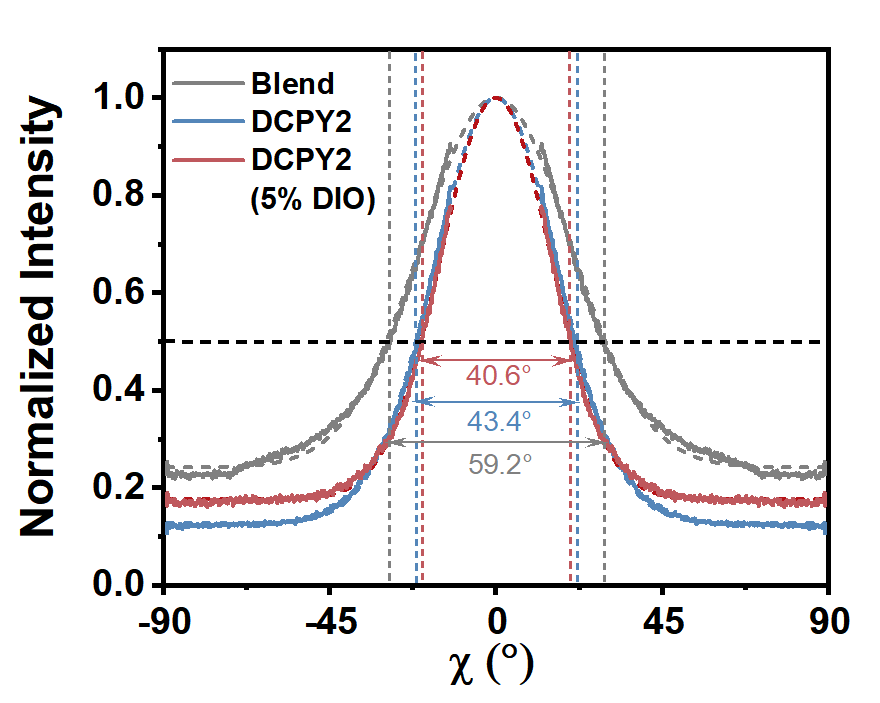


**Figure S22.** The azimuthal intensity plot of (010) peak in OOP direction of blend, DCPY2 and DCPY2 (5% DIO) films.


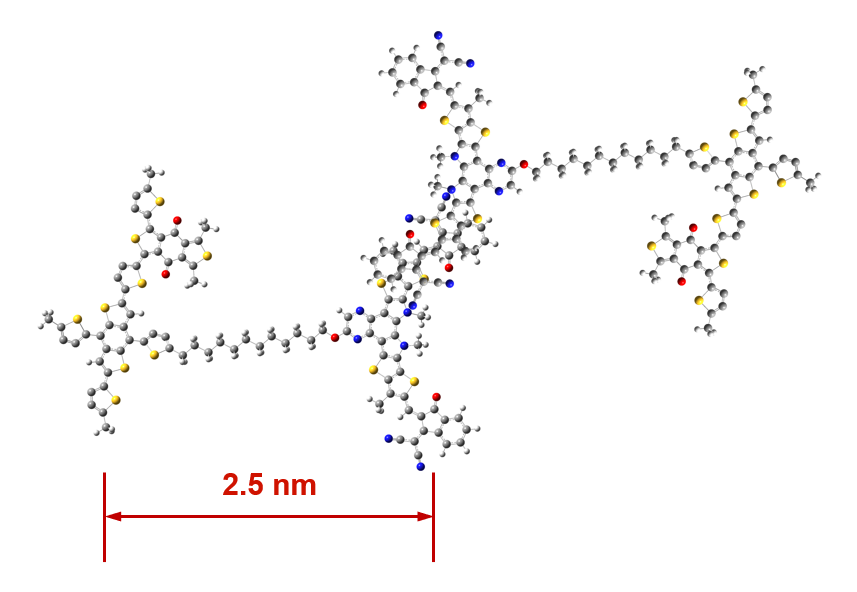


**Figure S23.** Structure of two neighboring DCPY2 molecule. The distance between a neighboring donor and acceptor is 2.5 nm and distance between neighboring donor is theoretically 5 nm. However, due to the curling of the long alkyl chain, this distance is supposed to be smaller than 5 nm, which is corresponding to the reasonable distance from GIWAXS data of 4.2 nm for distance between a neighboring donor segment.


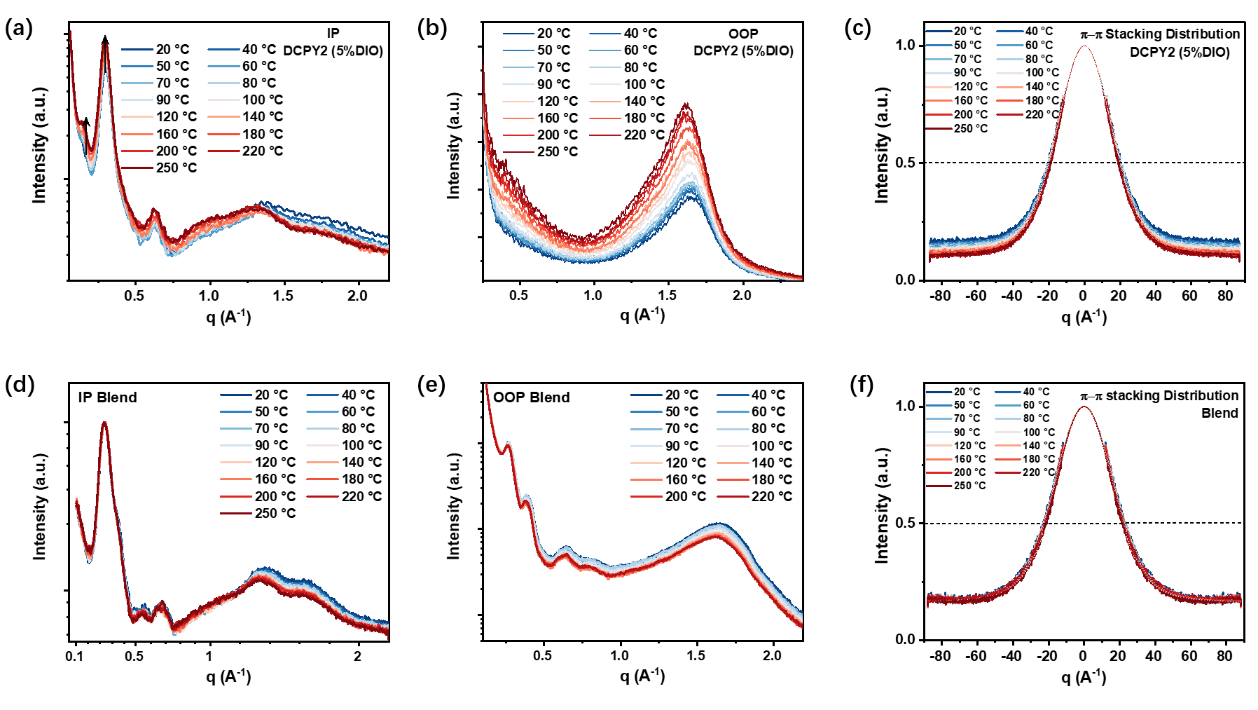


**Figure S24.** 1D linecuts of in-situ T-dependent GIWAXS of a,b) DCPY2 (5% DIO) and d,e) blend, in IP direction and OOP direction, respectively. The azimuthal intensity plot of (010) peak in OOP direction of c) DCPY2 (5% DIO) and f) blend. The thin Gaussian shape dash lines inside are the Lorentzian profile fitting for extrapolating the missing wedge.^[15]^ T-dependent GIWAXS indicted that DCPY2 (5% DIO) are more sensitive than blend, which is consistent with in-depth study of thermal driven phase separation by Feng et.al.^[16]^

# DFT calculation

**Figure S25.** Four possible dimers packing of Y-O6 calculated by DFT. Alkyl chains are simplified to methyl groups for calculation. According to their packing direction in DCPY2, it can be classified into 2 categories, intermolecular packing (containing dimer 1 and 2) and intramolecular packing (containing dimer 3 and 4).


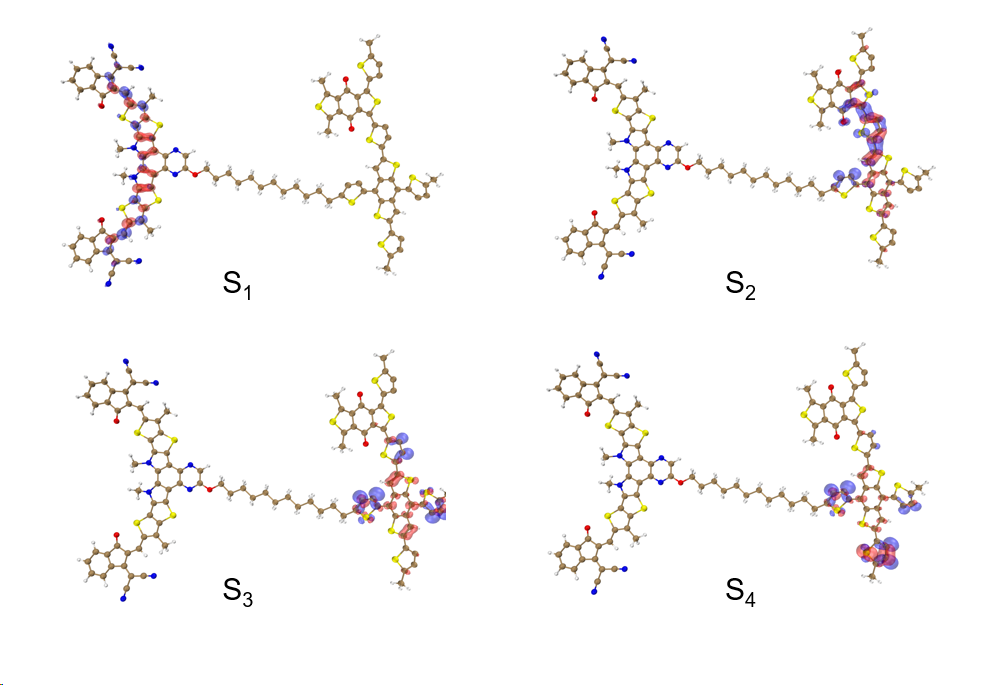


**Figure S26.** Charge density plots (red for holes and blue for electrons) of excited states in DCPY2.

**Table S1.** Photovoltaic performance of different devices under one sun illumination of solar simulator.

|  | ***J_SC_* (mA cm^-2^)** | ***J_Cal_* (mA cm^-2^)** | ***V_OC_* (V)** | **FF** | **PCE (%)** |
| --- | --- | --- | --- | --- | --- |
| Blend | 20.04 | 19.56 | 0.968 | 0.60 | 11.70 |
| DCPY2 | 21.16 | 20.64 | 0.934 | 0.61 | 12.10 |
| DCPY2 (5% DIO) | 23.04 | 22.39 | 0.911 | 0.66 | 13.85 |

**Table S2.** Summary of time constant of hole transfer process in TAS spectra (Figure 3c).

|  | ***A_1_* (%)** | $\boldsymbol{\tau}_{\boldsymbol{1}}$**(ps)** | ***A_2_* (%)** | $\boldsymbol{\tau}_{\boldsymbol{2}} \mathbf{(ps)}$ | $\boldsymbol{\tau}_{\boldsymbol{ave}}$**(ps)** |
| --- | --- | --- | --- | --- | --- |
| Blend | 28 | 1.1 | 72 | 14.8 | 11.0 |
| DCPY2 | 55 | 1.2 | 45 | 10.4 | 5.4 |
| DCPY2 (5% DIO) | 57 | 1.0 | 43 | 9.1 | 4.5 |

The hole transfer kinetics can be fitted by a biexponential function: $i=A_{1}exp(-t/\tau_{1})+ A_{2}exp(-t/\tau_{2})$.^[17]^ The hole transfer process consists of an ultrafast hole transfer process at the interface, as characterized by $\tau_{1}$, as well as a diffusion-mediated process strongly controlled by D/A domain size and aggregation, as characterized by $\tau_{2}$.

**Table S3.** Biexponential fitting results of TRPL decay with corresponding lifetimes $\tau_{1}$, $\tau_{2}$, effective average lifetime $\tau$.

|  | $\boldsymbol{\tau}_{\boldsymbol{1}}$**(ps)** | $\boldsymbol{\tau}_{\boldsymbol{2}} \mathbf{(ps)}$ | $\boldsymbol{\tau}\mathbf{(ps)}$ |
| --- | --- | --- | --- |
| Y-O6 | 881 | 479 | 560 |
| Blend | 29.46 | 77.33 | 43.54 |
| DCPY2 | 19.84 | 79.63 | 35.83 |
| DCPY2 (5% DIO) | 15.28 | 57.29 | 29.54 |

**Table S4.** Photovoltaic performance of different devices from TPV measurements.

|  | ***J_SC_* (mA cm^-2^)** | ***V*_OC_ (V)** | **FF** | **PCE (%)** |
| --- | --- | --- | --- | --- |
| Blend | 17.95 | 0.986 | 0.58 | 10.35 |
| DCPY2 | 17.33 | 0.914 | 0.6 | 9.97 |
| DCPY2 (5% DIO) | 20.26 | 0.889 | 0.63 | 11.29 |

**Table S5.** Difference in performance of previous DCP-based single-component system and binary system. The grey line indicates the performance of binary system.

|  | ***J_SC_* (mA cm^-2^)** | ***V_OC_* (V)** | **FF** | **PCE (%)** | ***E_CT_* (eV)** | **Reference** |
| --- | --- | --- | --- | --- | --- | --- |
| **As-DCPIC**  **(3% DIO)** | 21.23 | 0.77 | 0.62 | 10.09 | 1.43 | ^[18]^ |
| **PBDB-T:TPDIC** | 15.40 | 0.85 | 0.63 | 8.26 | 1.44 |  |
| **PBDBPBI-Cl** | 10.8 | 0.9 | 0.65 | 6.3 | - | ^[16]^ |
| **PBDB-T-Cl:PBI (0.5% DIO）** | 5.9 | 1.00 | 0.48 | 2.9 | - |  |
| **S-PBDTPBI** | 8.05 | 0.78 | 0.57 | 3.6 | - | ^[19]^ |
| **PBDTT:PBI** | 2.17 | 0.93 | 0.46 | 0.94 | - |  |
| **JP01** | 8.20 | 1.00 | 0.62 | 5.06 | - | ^[20]^ |
| **PBDB-T-Cl:NDI** | 1.53 | 1.12 | 0.43 | 0.74 | - |  |
| **JP02** **(2% DIO)** | 12.81 | 0.94 | 0.69 | 8.4 | - |  |
| **PClBDB-T:NDI** | 2.27 | 1.08 | 0.47 | 1.14 | - |  |

**Table S6.** Detailed analysis of *V*_OC_ shift from energetic contribution and kinetic contribution from TPV results.

|  | **Δ*V*_OC_**  **(mV)** | **Δ*V*_ele_ ^a^**  **(mV)** | **Δ*V*_kin_ ^b^**  **(mV)** | **Δ*V*_eff_ ^c^ (Δ*V*_ele_+Δ*V*_kin_)**  **(mV)** |
| --- | --- | --- | --- | --- |
| DCPY2 (5% DIO) - Blend | -97 | -214 | +112 | -102 |
| DCPY2 (5% DIO) - DCPY2 | -25 | -55 | +30 | -25 |
| DCPY2 - Blend | -72 | -159 | +82 | -77 |

^a^ Electronic bandgap shift (Δ*V_ele_*). ^b^ Kinetics Shift (Δ*V_kin_*). ^c^ Effective bandgap shift (Δ*V_eff_* =Δ*V_ele_*+Δ*V_kin_***)**.

Note that the device used for *V_OC_* analysis is not the same with best-performed one, but the trend is the same, which can give a hint for *V*_OC_ analysis.

**Table S7.** GISAXS parameters for PBDB-T, Y-O6, blend, DCPY2 and DCPY2 (5% DIO) films.

|  | **ξ (nm)** | **η (nm)** | **D** | **2R_g_ (nm)** |
| --- | --- | --- | --- | --- |
| Y-O6 | - | 13.1 | 3 | 64.1 |
| PBDB-T | 15.5 | 11.7 | 3 | 57.3 |
| Blend | 22.1 | 9.8 | 3 | 48 |
| DCPY2 | 17.3 | 6.3 | 3 | 30.8 |
| DCPY2 (5% DIO) | 19.3 | 7.4 | 3 | 36.3 |

**Table S8.** Detailed GIWAXS parameters for PBDB-T, Y-O6, blend, DCPY2 and DCPY2 (5% DIO) films in IP direction.

| Materials | lamellar stacking (100) in IP direction | | | | |
| --- | --- | --- | --- | --- | --- |
|  | Peak location [Å^-1^] | | d-space [Å] | FWHM of Peak [Å^-1^] | CCL^a^[Å] |
| PBDB-T | 0.3 | 20.93 | | 0.154 | 36.70 |
| Y-O6 | 0.36 | 17.44 | | - | - |
| Blend | 0.29 | 21.16 | | 0.088 | 64.26 |
|  | 0.38 | 16.53 | | 0.11 | 51.85 |
| DCPY2 | 0.15 | 51.87 | | 0.14 | 40.37 |
|  | 0.29 | 21.66 | | 0.07 | 80.74 |
|  | 0.36 | 17.44 | | 0.08 | 70.65 |
| DCPY2  (5% DIO) | 0.15 | 51.87 | | 0.08 | 70.65 |
|  | 0.29 | 21.66 | | 0.06 | 94.20 |
|  | 0.36 | 17.44 | | 0.10 | 56.52 |

^a^ Correlated cohesion length (CCL) = 0.9*2π/FWHM

**Table S9.** Detailed GIWAXS parameters for PBDB-T, Y-O6, blend, DCPY2 and DCPY2 (5% DIO) films in OOP direction.

| Materials | π-π stacking (010) in OOP direction | | | | |
| --- | --- | --- | --- | --- | --- |
|  | Peak location [Å^-1^] | d-space  [Å] | | FWHM of Peak [Å^-1^] | CCL [Å] |
| PBDB-T | 1.73 | | 3.63 | 0.42 | 13.46 |
| Y-O6 | 1.63 | | 3.85 | 0.52 | 10.87 |
| Blend | 1.63 | | 3.85 | 0.46 | 12.29 |
| DCPY2 | 1.63 | | 3.85 | 0.37 | 15.28 |
| DCPY2 (5% DIO) | 1.63 | | 3.85 | 0.29 | 19.49 |

**Table S10.** LUMO and HOMO level calculated by DFT of the four possible dimers packing of Y-O6.

|  | **LUMO (eV)** | **HOMO (eV)** | **HOMO-LUMO gap (eV)** |
| --- | --- | --- | --- |
| Dimer 1 | -3.493 | -5.535 | 2.042 |
| Dimer 2 | -3.554 | -5.453 | 1.899 |
| Dimer 3 | -3.493 | -5.535 | 2.042 |
| Dimer 4 | -3.446 | -5.486 | 2.040 |

# Supplementary Reference

[1] S. Liang, C. Xiao, C. Xie, B. Liu, H. Fang, W. Li, *Adv. Mater.* **2023**, 35, e2300629.

[2] J. Mai, T.-K. Lau, J. Li, S.-H. Peng, C.-S. Hsu, U. S. Jeng, J. Zeng, N. Zhao, X. Xiao, X. Lu, *Chem. Mater.* **2016**, 28, 6186.

[3] C. G. Shuttle, B. O’Regan, A. M. Ballantyne, J. Nelson, D. D. C. Bradley, J. de Mello, J. R. Durrant, *Appl. Phys. Lett.* **2008**, 92, 093311.

[4] M. J. Frisch, G. W. Trucks, H. B. Schlegel, G. E. Scuseria, M. A. Robb, J. R. Cheeseman, G. Scalmani, V. Barone, G. A. Petersson, H. Nakatsuji, X. Li, M. Caricato, A. V. Marenich, J. Bloino, B. G. Janesko, R. Gomperts, B. Mennucci, H. P. Hratchian, J. V. Ortiz, A. F. Izmaylov, J. L. Sonnenberg, Williams, F. Ding, F. Lipparini, F. Egidi, J. Goings, B. Peng, A. Petrone, T. Henderson, D. Ranasinghe, V. G. Zakrzewski, J. Gao, N. Rega, G. Zheng, W. Liang, M. Hada, M. Ehara, K. Toyota, R. Fukuda, J. Hasegawa, M. Ishida, T. Nakajima, Y. Honda, O. Kitao, H. Nakai, T. Vreven, K. Throssell, J. A. Montgomery Jr., J. E. Peralta, F. Ogliaro, M. J. Bearpark, J. J. Heyd, E. N. Brothers, K. N. Kudin, V. N. Staroverov, T. A. Keith, R. Kobayashi, J. Normand, K. Raghavachari, A. P. Rendell, J. C. Burant, S. S. Iyengar, J. Tomasi, M. Cossi, J. M. Millam, M. Klene, C. Adamo, R. Cammi, J. W. Ochterski, R. L. Martin, K. Morokuma, O. Farkas, J. B. Foresman, D. J. Fox, Wallingford, CT 2016.

[5] T. Lu, F. Chen, *J. Comput. Chem.* **2012**, 33, 580.

[6] W. Humphrey, A. Dalke, K. Schulten, *J. Mol. Graph.* **1996**, 14, 33.

[7] T. F. Hinrichsen, C. C. S. Chan, C. Ma, D. Palecek, A. Gillett, S. Chen, X. Zou, G. Zhang, H. L. Yip, K. S. Wong, R. H. Friend, H. Yan, A. Rao, P. C. Y. Chow, *Nat. Commun.* **2020**, 11, 5617.

[8] B. Sun, N. Tokmoldin, O. Alqahtani, A. Patterson, C. S. P. De Castro, D. B. Riley, M. Pranav, A. Armin, F. Laquai, B. A. Collins, D. Neher, S. Shoaee, *Adv. Energy Mater.* **2023**, 13, 2300980.

[9] T. Li, B. Li, H. Zhou, J. Wang, G. Ni, W. Ma, C. Sheng, J. Yuan, H. Zhao, *Adv. Funct. Mater.* **2024**, 34, 2311798.

[10] J. Bisquert, G. Garcia-Belmonte, *J. Phys. Chem. Lett.* **2011**, 2, 1950.

[11] C. G. Shuttle, A. Maurano, R. Hamilton, B. O’Regan, J. C. de Mello, J. R. Durrant, *Applied Physics Letters* **2008**, 93.

[12] C. G. Shuttle, R. Hamilton, J. Nelson, B. C. O'Regan, J. R. Durrant, *Advanced Functional Materials* **2010**, 20, 698.

[13] D. Credgington, J. R. Durrant, *J. Phys. Chem. Lett.* **2012**, 3, 1465.

[14] S. Wheeler, D. Bryant, J. Troughton, T. Kirchartz, T. Watson, J. Nelson, J. R. Durrant, *J. Phys. Chem. C* **2017**, 121, 13496.

[15] H.-C. Liao, C.-S. Tsao, Y.-T. Shao, S.-Y. Chang, Y.-C. Huang, C.-M. Chuang, T.-H. Lin, C.-Y. Chen, C.-J. Su, U. S. Jeng, Y.-F. Chen, W.-F. Su, *Energy Environ. Sci.* **2013**, 6, 1938.

[16] G. Feng, J. Li, Y. He, W. Zheng, J. Wang, C. Li, Z. Tang, A. Osvet, N. Li, C. J. Brabec, Y. Yi, H. Yan, W. Li, *Joule* **2019**, 3, 1765.

[17] Y. Zhong, M. T. Trinh, R. Chen, G. E. Purdum, P. P. Khlyabich, M. Sezen, S. Oh, H. Zhu, B. Fowler, B. Zhang, W. Wang, C. Y. Nam, M. Y. Sfeir, C. T. Black, M. L. Steigerwald, Y. L. Loo, F. Ng, X. Y. Zhu, C. Nuckolls, *Nat. Commun.* **2015**, 6, 8242.

[18] S. Liang, B. Liu, S. Karuthedath, J. Wang, Y. He, W. L. Tan, H. Li, Y. Xu, N. Li, J. Hou, Z. Tang, F. Laquai, C. R. McNeill, C. J. Brabec, W. Li, *Angew. Chem., Int. Ed.* **2022**, 61, e202209316.

[19] G. Feng, J. Li, F. J. M. Colberts, M. Li, J. Zhang, F. Yang, Y. Jin, F. Zhang, R. A. J. Janssen, C. Li, W. Li, *J. Am. Chem. Soc.* **2017**, 139, 18647.

[20] X. Jiang, J. Yang, S. Karuthedath, J. Li, W. Lai, C. Li, C. Xiao, L. Ye, Z. Ma, Z. Tang, F. Laquai, W. Li, *Angew. Chem., Int. Ed.* **2020**, 59, 21683.
